# Mitogen-Activated Protein Kinase OsMEK2 and OsMPK1 Signaling Is Required for Ferroptotic Cell Death in Rice–*Magnaporthe oryzae* Interactions

**DOI:** 10.3389/fpls.2021.710794

**Published:** 2021-08-02

**Authors:** Sarmina Dangol, Nam Khoa Nguyen, Raksha Singh, Yafei Chen, Juan Wang, Hyeon-Gu Lee, Byung KooK Hwang, Nam-Soo Jwa

**Affiliations:** ^1^Division of Integrative Bioscience and Biotechnology, College of Life Sciences, Sejong University, Seoul, South Korea; ^2^Department of Plant Physiology, Swammerdam Institute for Life Sciences, University of Amsterdam, Amsterdam, Netherlands; ^3^Crop Production and Pest Control Research Unit, United States Department of Agriculture-Agricultural Research Service, Purdue University, West Lafayette, IN, United States; ^4^Division of Biotechnology, College of Life Sciences and Biotechnology, Korea University, Seoul, South Korea

**Keywords:** cell death, ferroptosis, iron, *Magnaporthe oryzae*, mitogen-activated protein kinase (MAPK) signaling, reactive oxygen species (ROS), rice

## Abstract

Mitogen-activated protein kinase (MAPK) signaling is required for plant cell death responses to invading microbial pathogens. Iron- and reactive oxygen species (ROS)-dependent ferroptotic cell death occurs in rice (*Oryza sativa*) during an incompatible rice–*Magnaporthe oryzae* interaction. Here, we show that rice MAP kinase (OsMEK2 and OsMPK1) signaling cascades are involved in iron- and ROS-dependent ferroptotic cell death responses of rice to *M. oryzae* infection using *OsMEK2* knock-out mutant and *OsMEK2* and *OsMPK1* overexpression rice plants. The OsMPK1:GFP and OsWRKY90:GFP transcription factor were localized to the nuclei, suggesting that OsMPK1 in the cytoplasm moves into the nuclei to interact with the WRKY90. *M. oryzae* infection in Δ*Osmek2* knock-out plants did not trigger iron and ROS accumulation and lipid peroxidation, and also downregulated *OsMPK1, OsWRKY90, OsRbohB*, and *OsPR-1b* expression. However, 35S:*OsMEK2* overexpression induced ROS- and iron-dependent cell death in rice. The downstream MAP kinase (*OsMPK1*) overexpression induced ROS- and iron-dependent ferroptotic cell death response to virulent *M. oryzae* infection. The small-molecule ferroptosis inhibitor ferrostatin-1 suppressed iron- and ROS-dependent ferroptotic cell death in 35S:*OsMPK1* overexpression plants. However, the small-molecule inducer erastin triggered iron- and lipid ROS-dependent, but *OsMEK2*-independent, ferroptotic cell death during *M. oryzae* infection. Disease (susceptibility)-related cell death was lipid ROS-dependent, but iron-independent in the Δ*Osmek2* knock-out mutant during the late *M. oryzae* infection stage. These combined results suggest that *OsMEK2* and *OsMPK1* expression positively regulates iron- and ROS-dependent ferroptotic cell death, and blast disease (susceptibility)-related cell death was ROS-dependent but iron-independent in rice–*M. oryzae* interactions.

## Introduction

Plants have evolved effective innate immune system responses to avert the invasion of microbial pathogens in their natural habitat (Dodds and Rathjen, 2010; Schwessinger and Ronald, 2012; Fu and Dong, 2013). Plant immune system responses are mediated by pattern-triggered immunity (PTI) and effector-triggered immunity (ETI), which are effectively upregulated inside plant cells in response to pathogen infection (Jones and Dangl, 2006). PTI is activated by plant perception of conserved microbial structures, called pathogen-associated molecular patterns (PAMPs), via the transmembrane pattern recognition receptors (PRRs) (Zipfel, 2008). ETI is activated by plant recognition of specific pathogen effector molecules via intracellular nucleotide-binding leucine-rich repeat (NLR) receptors, called resistance (R) proteins (Jones and Dangl, 2006; Block and Alfano, 2011; Oh and Martin, 2011). The two immune systems trigger a series of molecular signaling events that lead to diverse cellular responses including transcriptional reprogramming, synthesis of defense-related proteins, reactive oxygen species (ROS) burst, and iron- and ROS-dependent ferroptotic cell death (Boller and He, 2009; Dangol et al., 2019).

Mitogen-activated protein (MAP) kinase (MAPK) signaling pathways have pivotal roles in plant defense, immunity, and hypersensitive cell death responses to pathogen attack (Ishihama et al., 2011; Meng and Zhang, 2013; Thulasi Devendrakumar et al., 2018). However, the downstream signaling networks activated by defense-related MAPKs have not been completely defined in plants. Plant MAPK cascades proceed through three central kinases: MAPK kinase kinase (MAPKKK); MAPK kinase (MAPKK), also known as MAPK and ERK (extracellular signal-regulated kinase) kinase (MEK); and MAP kinase (MAPK or MPK) (Meng and Zhang, 2013). These kinases are sequentially phosphorylated as MAPKKKs activate downstream MAPKKs (MEKs), which subsequently activate MAPKs (Rodriguez et al., 2010). Phosphorylation of MAPKs may promote their nuclear translocation to target other kinases, proteins, or transcription factors in the nucleus (Khokhlatchev et al., 1998; Rodriguez et al., 2010). MAPKs can activate transcription factors such as WRKYs. The Arabidopsis genome encodes 60 MAPKKKs, 10 MAPKKs, and 20 MAPKs (Ichimura et al., 2002). A previous study showed that Arabidopsis innate immune responses are mediated by a MAP kinase signaling cascade (MEKK1, MKK4/MKK5, and MPK3/MPK6) and WRKY22/WRKY29 transcription factors (Asai et al., 2002). Arabidopsis MPK3 and MPK6 are involved in ETI (Tsuda et al., 2009; Meng and Zhang, 2013; Su et al., 2018). Pathogen-responsive MAPK cascades (MEKK1-MKK4/MKK5-MPK3/MPK6 and MEKK1-MKK1/2-MPK4) have pivotal roles in defense signaling against pathogen attack in *Arabidopsis thaliana* (Pitzschke et al., 2009; Rasmussen et al., 2012; Meng and Zhang, 2013). Nearly two decades ago, Yang et al. (2001) identified a tobacco MAPKK (NtMEK2) upstream of both salicylic acid-induced protein kinase (SIPK) and wounding-induced protein kinase (WIPK). Expression of a constitutively active *NtMEK2* mutant induced hypersensitive response (HR)-like cell death and defense responses in tobacco. Many kinases in MAPK cascades, including MAPKKK, MEK, SIPK/WIPK, and MAPK, are involved in *N* gene-mediated resistance to tobacco mosaic virus in tobacco (Jin et al., 2002, 2003; Liu et al., 2004). Tobacco WRKY/MYB transcription factors downstream of MAPK cascades have crucial roles in regulating *N*-mediated resistance to TMV (Liu et al., 2004). Silencing of *MEK2* (*SlMKK2*), *SlMPK2*, and *SlMKK4* in tomato disrupted the resistance to infection by *Xanthomonas campestris* pv. *vesicatoria* (*Xcv*) and *Botrytis cinerea* (Melech-Bonfil and Sessa, 2011; Li et al., 2014).

The rice genome contains 74 MAPKKK, 8 MAPKK, and 17 MAPK genes (Hamel et al., 2006; Reyna and Yang, 2006; Rao et al., 2010; Yang et al., 2015). We previously identified 74 non-redundant interactors with rice MAPKs and performed high-resolution mapping of the MAPK interactome network, which controls different signaling pathways underlying the cellular and physiological responses in rice (Singh et al., 2012). Rice MAP kinase kinase1 (OsMEK1) physically interacts with rice MAP kinase1 (OsMPK1), OsMPK6, and OsMPK5. OsMEK2 interacts with OsMPK1 and OsMPK6. OsMEK6 interacts with OsMPK1 and OsMPK5 (Singh et al., 2012). Rice MAP kinase kinases (OsMAP2Ks or OsMEKs) may regulate multiple signaling pathways affecting many biological processes by associating with different sets of rice MAPK interactomes (Singh and Jwa, 2013). However, few kinase components in rice MAPK cascades are involved in immunity and defense responses in rice–pathogen interactions (Singh and Jwa, 2013; Yang et al., 2015). OsMKK10-2–mediated activation of OsMPK6 via specific phosphorylation subsequently induced *WRKY45* expression and blast (*Magnaporthe oryzae*) resistance in rice plants (Ueno et al., 2015). OsMPKK10-2 is involved in disease resistance and drought tolerance (Ma et al., 2017), physically interacts with OsMPK6 and OsMPK3, and phosphorylates the two OsMPKs, leading to *X. oryzae* pv. *oryzicola* (*Xoo*) resistance and drought tolerance (Ma et al., 2017). The MAP kinase module OsMKK3-OsMPK7-OsWRKY30 is involved in induced resistance to *Xanthomonas oryzae* pv. *oryzicola* (*Xoo*) infection in rice (Jalmi and Sinha, 2016).

Cell death is a fundamental biological process that occurs during development, senescence, immunity, and stress resistance in multicellular organisms. ROS bursts are involved in basal immune responses, NLR-mediated hypersensitive cell death, and disease-associated cell death in plants (Greenberg and Yao, 2004; Choi et al., 2012, 2013; Jwa and Hwang, 2017). Ferroptosis differs from apoptosis, necrosis, and autophagy, and was first discovered in mammalian cells as a form of non-apoptotic iron-dependent cell death (Dixon et al., 2012). Ferroptotic cell death requires the accumulation of ROS, iron, and lipid peroxides (Stockwell et al., 2017). Iron homeostasis and ROS burst have important roles in activating defense responses against plant pathogens (Liu et al., 2007; Aznar et al., 2015). We recently reported that iron- and ROS-dependent ferroptosis occurs in rice–*Magnaporthe oryzae* interactions (Dangol et al., 2019). This is the first plant pathosystem in which ferroptotic cell death was discovered (Caseys, 2019). Avirulent *M. oryzae* infection triggers iron and ROS (H_2_O_2_) accumulation at the cell death sites in rice tissues (Dangol et al., 2019). Iron is required for lipid peroxide accumulation. Iron and ROS accumulation and lipid peroxidation are blocked by the iron chelator deferoxamine, the lipophilic antioxidant ferrostatin-1, the actin polymerization inhibitor cytochalasin E, and the NADPH-oxidase inhibitor diphenyleneiodonium (DPI), thereby restricting HR cell death in rice (Dangol et al., 2019). By contrast, the RAS-selective lethal small molecule inducer erastin triggered iron-dependent ROS accumulation and glutathione depletion, which ultimately promoted *M. oryzae-*induced ferroptotic cell death. Rice NADP-malic enzyme (NADP-ME) and NADPH-oxidase (Rboh) are ROS sources that have been proposed to be involved in iron- and ROS-dependent ferroptotic cell death (Singh et al., 2016; Dangol et al., 2019).

Our previous study identified rice mitogen-activated protein (MAP) kinase kinase 2 (OsMEK2) as a rice MAP interactor (Singh et al., 2012). Rice MAP kinase (OsMPK1) is an interactor of OsMEK2 and actively involved in *M. oryzae* infection (Singh et al., 2012; Ueno et al., 2015). Here, we used *OsMEK2* and *OsMPK1* to investigate whether rice MAPKs are involved in the signaling network that mediates ferroptotic cell death in rice–*M. oryzae* interactions. *OsMEK2* knock-out via T-DNA insertion in rice cultivar Dongjin (DJ) suppressed iron- and ROS-dependent ferroptotic cell death, which ultimately induced susceptible responses to avirulent *M. oryzae* 007 infection. However, OsMEK2 overexpression in rice DJ induced iron- and ROS-dependent ferroptotic cell death against *M. oryzae* 007 infection. Treatment of the *OsMEK2*-knockout mutants with erastin induced the ROS burst and iron accumulation, which caused ferroptotic cell death in Δ*Osmek2* knock-out plants in response to *M. oryzae* infection. Disease (susceptibility)-related cell death at the late stage of *M. oryzae* infection in Δ*Osmek2* knock-out plants is ROS-dependent and iron-independent. During *M. oryzae* infection, *OsMEK2* knock-out and overexpression differentially regulated the expression of *OsMPK1*, *OsMPK6*, and the *OsWRKY90* transcription factor in the rice MAPK signaling pathways. *OsMPK1* overexpression in susceptible rice cultivar Nipponbarre (NB) induced iron- and ROS-mediated ferroptotic cell death against *M. oryzae* PO6-6 infection. Treatment with ferrostatin-1 suppressed iron- and ROS-dependent ferroptotic cell death in 35S:*OsMPK1* overexpression leaf sheaths during infection. These combined results indicate that *OsMEK2* and *OsMPK1* expression via MAPK signaling pathway positively regulates iron- and ROS-dependent ferroptotic cell death and plant immunity to *M. oryzae* infection.

## Materials and Methods

### Plant Materials and Growth Conditions

The WT rice cultivars Dongjin (DJ) and Nipponbarre (NB) and the Δ*Osmek2* knock-out, 35S:*OsMEK2* and 35S:*OsMPK1* overexpression lines were used in this study. Δ*Osmek2* T-DNA insertion knock-out mutant seeds were provided by the Rice Functional Genomic Express Database (RiceGE) managed by the Salk Institute Genomic Analysis Laboratory^1^ (Jeon et al., 2000). DJ and NB rice seeds were obtained from the National Institute of Crop Science, South Korea^2^. Rice seeds were germinated in water for 5 days and then planted in plastic pots containing Baroker soil (Seoul Bio, South Korea). Rice plants were grown in growth chambers at 28°C under white fluorescent light (150 μmol photons m^–2^ s^–1^) with a 16 h photoperiod and 60% relative humidity, as described previously (Dangol et al., 2019).

### Fungal Cultures and Growth Conditions

The rice blast fungal strains *Magnaporthe oryzae* 007 and PO6-6 were provided by the Center for Fungal Genetic Resources, Seoul National University, Seoul, South Korea^3^. *M*. *oryzae* 007 was avirulent (incompatible) and *M*. *oryzae* PO6-6 was virulent (compatible) to the rice cultivar DJ. The rice cultivar NB was susceptible to *M*. *oryzae* PO6-6 infection. The fungal cultures were stored at –20°C and cultured on rice bran agar media (20 g rice bran, 20 g sucrose, and 20 g agar in 1 L Milli-Q water). *M. oryzae* strains were grown at 25°C in the dark for 2 weeks. *M. oryzae* sporulation was induced by removing aerial mycelia from the fungal culture plates, followed by their incubation under a continuous fluorescent light (80 μmol photons m^–2^ s^–1^) for 2–3 days at 25°C.

### Fungal Inoculation of Rice Tissues and Infection Evaluation

Conidial suspensions of *M. oryzae* strains were inoculated on rice leaves and leaf sheaths as described previously (Singh et al., 2016; Dangol et al., 2019). *M. oryzae* conidia were harvested from the sporulated culture plates using a 0.025% Tween 20 (Sigma-Aldrich) solution. The conidial concentration was adjusted to 4 × 10^5^ conidia mL^–1^. The conidial suspension was spray-inoculated over the surface of 2-week-old rice seedlings. The inoculated seedlings were incubated at 25–28°C for 24 h under dark and moist conditions, and then were moved to normal conditions (16 h light/8 h dark). Disease phenotypes were observed at 5 days after inoculation and classified with respect to susceptible (large, elliptical, grayish, and expanded lesions) and resistant (slightly elongated, necrotic brownish spots) reactions.

Middle-aged leaf sheaths (5–7 cm lengths) of 4- or 5-week-old rice plants were inoculated with *M*. *oryzae* conidial suspensions (4 × 10^5^ conidia mL^–1^). Inoculated leaf sheaths were incubated in a moistened box with 100% relative humidity at 25°C under dark conditions. After incubation for different times, epidermal layers were excised from the leaf sheaths, cut into 1.5 cm lengths, and fixed on glass microscope slides. The infected epidermal cells from each of three epidermal sheaths were observed under the microscope and divided into two infection phenotypes: cells with viable IH and cells with HR cell death. For the quantification of invasive hyphae (IH) and HR cell death, approximately 500 infected cells in each of the leaf sheaths were counted at least three times from one representative of three independent experiments.

### Identification of T-DNA Insertion in Δ*Osmek2* Mutants

Δ*Osmek2* T-DNA insertion mutant seeds from RiceGE (Jeon et al., 2000) were screened by PCR using the left gene-specific primer (LP), the right gene-specific primer (RP), and the T-DNA right border primer (RB). The gene-specific primers are listed in Supplementary Table 1. To verify homozygosity in the knock-out mutant plants, PCR was performed with LP and RP primers of the gene, and homozygous plants were identified by the lack of specific PCR products. The LP and RB primers were used for PCR analysis to confirm the presence of the T-DNA insertion in Δ*Osmek2* knock-out mutant plants. Quantitative real-time RT-PCR and immunoblotting analyses were performed to verify whether transcriptional and translational expression of *OsMEK2* were blocked in Δ*Osmek2* knock-out mutants.

### Rice Transformation

The full length cDNAs of *OsMEK2* and *OsMPK1* were amplified from rice cDNA library and inserted into plant expression vector pCAMLA under the control of CaMV 35S promoter, followed by selection of hygromycin gene (*hph*). The constructed CaMV 35S:*OsMEK2* and CaMV 35S:*OsMPK1* were introduced in the rice cultivars DJ and NB, respectively, by *Agrobacterium tumefaciens-*mediated transformation, as described previously with slight modification (Hiei et al., 1994).

Briefly, 35S:*OsMEK2* and 35S:*OsMPK1* were delivered into rice calli using *A. tumefaciens* strain LBA4404 (Hoekema et al., 1983; Lee et al., 2005). The transformed calli were selected on the selection media containing gradually increasing concentrations of hygromycin (30 mg/L and 60 mg/L). After rooting and shooting, rice seedlings were transferred in water for 4 days. After adaption in water, rice seedlings were raised in soil in a growth chamber. The positive transformants from T_0_ generation were selected by PCR using hygromycin primers. Next, seeds of T_1_ generation were analyzed on the hygromycin-containing media and T_2_ generation seeds were used for homozygote selection. The functional analysis was performed from T_3_ generation. The levels of gene expression were determined by immunoblot analysis and qRT-PCR. The primers used for the experiments are listed in Supplementary Table 1.

### Real-Time RT-PCR Analyses

Total RNA was isolated from rice tissue using TRIzol reagent (Invitrogen) according to the manufacturer’s instructions. The gene expression levels were analyzed by reverse-transcription PCR (RT-PCR) and real-time quantitative PCR (qRT-PCR). First-strand cDNA was synthesized from 2 μg total RNA in 20 μL reaction mixture using a cDNA synthesis kit (Invitrogen) according to the manufacturer’s instructions. Prepared cDNA (1 μL) was used as a template for both RT-PCR and qRT-PCR. The qRT-PCR was performed using TOPreal^TM^ qPCR 2 × PreMIX (SYBR Green with low ROX; Enzynomics, Daejeon, South Korea) according to the manufacturer’s instructions. Relative gene expression levels were determined using rice 18S ribosomal RNA or rice ubiquitin as an internal standard gene. Gene-specific primers used for the real-time RT-PCR analysis are listed in Supplementary Table 1.

### Protein Isolation and Immunoblot Assay

Rice proteins were extracted using trichloroacetic acid (TCA)/acetone extraction buffer [TCAAEB; 10% (w/v) trichloroacetic acid and 0.07% β-mercaptoethanol in 100 mL acetone] as described previously (Cho et al., 2006). Rice leaves were ground to a fine powder using liquid nitrogen in a mortar and pestle. Then, proteins were precipitated with TCAAEB and washed three times with wash buffer [0.07% β-mercaptoethanol, 2 mM ethylenediaminetetraacetic acid (EDTA), and EDTA-free protease inhibitor cocktail tablet (Roche) in a final volume of 100 mL acetone]. Protein precipitates were air-dried at room temperature, stored at –80°C for at least 24 h, and solubilized in lysis buffer containing thiourea and Tris (LB-TT) {7 M urea, 2 M thiourea, 4% (w/v) 3-[(3-cholamidopropyl) dimethylammonio]-l-propanesulfonate (CHAPS), 18 mM Tris-HCI (pH 8.0), 14 mM Trizma base, two EDTA-free protease inhibitor cocktail tablets (Roche), 0.2% (v/v) Triton X-100, and 50 mM dithiothreitol (DTT) in a final volume of 100 mL}. After centrifuging at 15,000 × *g* for 15 min at 4°C, the supernatants were precipitated using pre-chilled acetone and solubilized in LB-TT buffer.

The OsMEK2 protein expression levels in DJ and Δ*Osmek2* mutant plants were determined by 10% SDS-polyacrylamide gel electrophoresis (PAGE) followed by immunoblot analysis using rabbit polyclonal anti-MEK2 antibody (EnoGene^®^ E580135-A-SE). Immuno-reactive target bands were detected by Odyssey^®^ CLx Imaging System (LI-COR Biosciences). Equal gel loading was checked by Ponceau S staining.

### Erastin and Ferrostatin-1 Treatment

The small molecule cell death inducer erastin was used to investigate whether erastin treatment triggered ferroptotic cell death in rice leaf sheath cells as described previously (Dangol et al., 2019). *M. oryzae* conidia (4 × 10^5^ conidia mL^–1^) were mixed with 10 μM erastin (Sigma-Aldrich, St. Louis, MO, United States) and inoculated on leaf sheaths. The erastin-treated and *M. oryzae*-inoculated leaf sheath tissues were incubated in the dark at 25°C. The ferroptosis inhibitor, ferrostatin-1 (Fer-1, Sigma-Aldrich), was treated as described previously (Dangol et al., 2019). Rice epidermal layers were excised from the *M. oryzae-*infected leaf sheaths and then vacuum-infiltrated in 10 μM Fer-1 solution for 10 min, followed by their incubation for 24 h.

### CM-H_2_DCFDA Assay and DAB Staining

Cellular ROS (H_2_O_2_) localization in rice leaf sheath cells was visualized using 5- (and 6-) chloromethyl-2′,7′-dichlorofluorescin diacetate acetyl ester (CM-H_2_DCFDA) and 3,3′-diaminobenzidine (DAB) staining methods as described previously (Shin et al., 2005; Dangol et al., 2019). Briefly, thin epidermal layers of rice leaf sheaths were excised and cut into equal pieces, followed by incubation in 1 mL water for 5 min to remove wound-induced ROS. Epidermal sheath samples were incubated in 2 μM CM-H_2_DCFDA (Molecular Probes Life Technologies, Eugene, OH, United States) in 1 × phosphate-buffered saline (PBS) buffer in the dark for 30 min on a horizontal shaker. The incubated sheath samples were washed twice with 1 × PBS buffer for 5 min in the dark. ROS localization inside the epidermal sheath cells was observed immediately under a fluorescence microscope.

For DAB staining, epidermal layers of rice leaf sheaths were vacuum-infiltrated with 1 mg mL^–1^ DAB (Sigma-Aldrich, St. Louis, MO, United States) solution for 5 min, followed by overnight destaining with ethanol:acetic acid:glycerol (3:1:1, v/v/v). ROS localization in the DAB-stained epidermal cells was observed under a microscope. The DAB-stained cells were categorized into two phenotypes: Type I, infected cells that display no or weak DAB staining; and Type II, infected cells that display strong DAB staining. DAB-stained 500 cells with different phenotypes were counted from each of infected sheaths. The counted cell numbers were then converted into the percentages of DAB-stained cells.

### Chemiluminescence Assay for ROS Measurement

The chemiluminescence assay was used to measure ROS production in rice leaf sheaths as described previously (Singh et al., 2016; Dangol et al., 2019) with minor modifications. Epidermal layers of treated and *M. oryzae*-inoculated rice leaf sheaths were cut into small pieces (0.5 cm length) and incubated in 1 mL of sterilized Milli-Q water for 5 min to remove wound-induced ROS. Then, a piece of epidermal layer was added into a mixture of 30 μL luminol (Bio-Rad, Hercules, CA, United States), 1 μL horseradish peroxidase (Jackson Immunoresearch, West Grove, PA, United States), and 69 μL Milli-Q water in each well of a 96-well plate. Chemiluminescence (RLU, relative luminescent units) was detected from the ROS signals after 5 min incubation using a GloMax^®^ 96 Microplate Luminometer (Promega, Madison, WI, United States).

### Malondialdehyde (MDA) Assay

The malondialdehyde (MDA) assay was performed to determine lipid peroxidation in rice leaf sheath tissues as described previously (Zhang et al., 2009; Dangol et al., 2019). Lipid peroxidation is the degradation of lipids due to oxidative damage in plant cells. Briefly, the ground tissue powder of rice leaf sheath was mixed with the reaction solution [0.5% (w/v) thiobarbituric acid, 20% (v/v) trichloroacetic acid (TCA), and 0.25 mL 175 mM NaCl in 2 mL of 50 mM Tris-Cl, pH 8.0]. The mixed reaction was then incubated in boiling water for 5 min, cooled on ice for 5 min, and centrifuged at 14,000 × *g* for 5 min. The MDA concentration (C) in the resulting supernatant was determined by measuring supernatant absorbances (OD, optical density) at 450, 532, and 600 nm, and then calculating MDA concentration according to the following equation: *C* = 6.45 × (OD_532_–OD_600_) – (0.56 × OD_450_).

### Ferric Ion (Fe^3+^) Detection by Prussian Blue Staining

Prussian blue staining was performed to detect ferric ion (Fe^3+^) in rice leaf sheaths as described previously (Liu et al., 2007; Dangol et al., 2019). Briefly, epidermal layers of rice leaf sheaths were excised and incubated in equal volumes (1:1, v/v) of 7% potassium ferrocyanide and 2% hydrochloric acid (HCl) for 15 h at room temperature. Prussian blue (ferric ferrocyanides, which combine with Fe^3+^ inside leaf sheath epidermal cells) was observed as a bright blue color under a fluorescence microscope. Prussian blue-stained cells were categorized into two phenotypes: Type I, cells that contain IH but are weakly or not Prussian blue-stained; and Type II, strongly Prussian blue-stained cells with only a few poor hyphae. Prussian blue-stained 500 cells with different phenotypes were counted from each of infected sheaths. The counted cell numbers were then converted into the percentages of Prussian blue-stained cells.

### Subcellular Localization of OsMEK2, OsMPK1, and OsWRKY90 in *N. benthamiana* Leaves

The full length cDNA of *OsMEK2*, *OsMPK1*, and *OsWRKY90* were amplified from the rice cDNA library with the gene-specific primers containing attB1 and attB2 sites, as described in Supplementary Table 1. The amplified PCR products were used as a template for the second PCR using attB1 and attB2 primers. The second PCR products were sub-cloned into the pDONR^TM^201 entry vector using BP clonase (Invitrogen) to create entry clones. The entry clones were recombined into the Gateway binary vector pGWB552 tagged with G3 green fluorescent protein (G3GFP) using LR clonase (Invitrogen) (Nakagawa et al., 2007; Dangol et al., 2017).

The binary plasmids containing *OsMEK2*, *OsMPK1*, and *OsWRKY90* were transformed into *A. tumefaciens* GV3101. Recombinant agrobacteria were prepared for infiltration using a protocol as described previously with slight modification (Sainsbury and Lomonossoff, 2008). Briefly, single colonies of recombinant agrobacteria were incubated into the liquid LB media (10 g/L tryptone, 5 g/L yeast extract; 10 g/L NaCl, pH 7) containing spectinomycin (100 μg/L) for overnight at 28°C with continuous shaking. Harvested recombinant agrobacteria were resuspended to an OD_600_ = 0.2 in MMA (10 mM MES pH 5.6, 10 mM MgCl_2_, 150 μM acetosyringone). The agrobacterial suspension was incubated for 2 h at room temperature, and infiltrated into the abaxial leaves of 6-week-old *Nicotiana benthamiana* plants using a blunt tipped plastic syringe. Subcellular localization of 00:GFP, OsMEK2:GFP, OsMPK1:GFP, and OsWRKY90:GFP in *N. benthamiana* epidermal cells 36 h after agroinfiltration were microscopically observed following 4′,6-diamidino-2-phenylindole (DAPI, 5 μg/ml) staining for 10 min. Nuclear localization of the proteins was visualized by counterstaining the nuclei of the cells with DAPI.

### Microscopy

Images were captured using a fluorescence microscope (Zeiss equipped with Axioplan 2; Campbell, CA, United States) with 40× oil-immersion objective lens. CM-H_2_DCFDA-specific fluorescence was visualized under the fluorescence microscope using a combination of excitation (450–490 nm) and emission (515–565 nm) green fluorescence (GF) filters. Subcellular images were also taken using a fluorescence microscope (Olympus, Japan) using bright field, GF (Ex/Em: 488/498–548 nm), and DAPI (Ex/Em: 405/421–523 nm) filters.

### Accession Numbers

Sequence data from this article can be found in the GenBank/EMBL data libraries under the following accession numbers: *OsMEK1* (Os01g32660), *OsMEK2* (Os06g05520), *OsMEK3* (Os03g12390), *OsMEK4* (Os02g46760), *OsMEK5* (Os06g09190), *OsMEK6* (Os02g54600), *OsMEK7* (Os06g09180), *OsMEK8* (Os06g27890), *OsMPK1* (Os06g06090), *OsMPK6* (Os10g38950), *OsWNK1* (Os07g38530), *OsWRKY90* (Os09g30400), *OsNADP-ME* (Os01g52500), *OsRbohB* (Os01g25820), *OsPR-1b* (Os01g28450), *OsPAL1* (Os04g43760), *OsAPX1* (*Os0.g17690*), *OsAPX2* (*Os07g49400*), *OsUbiqutin* (Os06g46770), *18S rRNA* (XR_003238819.1), *AtMKK1* (At4g26070), *AtMKK2* (At4g29810), *AtMEK3* (NP_198860), *AtMEK4* (At1g51660), *AtMEK5* (At3g21220), *AtMKK6* (At5g56580), *AtMKK9* (At1g73500), *AtWNK9* (At3g04910), *NtNPK2* (BAA06731), *SlMKK1* (NP_001234744), *SlMKK2* (NP_001234588), *NbMEK2* (LOC107818847), and *NtMEK2* (AF325168).

## Results

### Identification of the Rice MAPK Interactor OsMEK2

In our previous study, we isolated rice mitogen-activated protein kinase kinases (MAPKKs or MEKs) that interacted with OsMAPKs using yeast two-hybrid analysis (Singh et al., 2012). Amino acid sequences of the isolated rice MEKs were aligned with those of Arabidopsis AtMAPKKs, and subsequently categorized into Groups A–D (Ichimura et al., 2002) (Supplementary Figure 1). The plant MAPKKs contained 11 conserved subdomains (Supplementary Figure 2). All of the aligned MAPKKs contained the active site domain [D(I/L/V)KP] and the conserved motif (S/T-X_5_-S/T, where X represents any amino acid residue) (Supplementary Figure 1A). The rice MAPKKs OsMEK1 and OsMEK2 belonged to Group A serine (S)/threonine (T) kinases. OsMEK1 amino acid sequence shared 54% and 53% homology with AtMKK1 and AtMKK2, respectively, whereas OsMEK2 had 62% sequence homology with both AtMKK1 and AtMKK2. A phylogenetic tree was generated to compare OsMEKs with Arabidopsis MAPKKs (Kumar et al., 2016) (Supplementary Figure 1B). OsMEK2 shares 65–66% homology with *N. benthamiana* NbMEK2 and tomato SlMKK1 (Supplementary Figure 2). OsMEK2 was phylogenetically close to NbMEK2, SlMKK1, AtMKK1 and AtMKK2 (Supplementary Figure 3). Based on the sequence alignment data of rice MAPKKs, OsMEK2 was selected to investigate whether rice MAPKKs are required for ferroptotic cell death signaling in this study.

**FIGURE 1:**
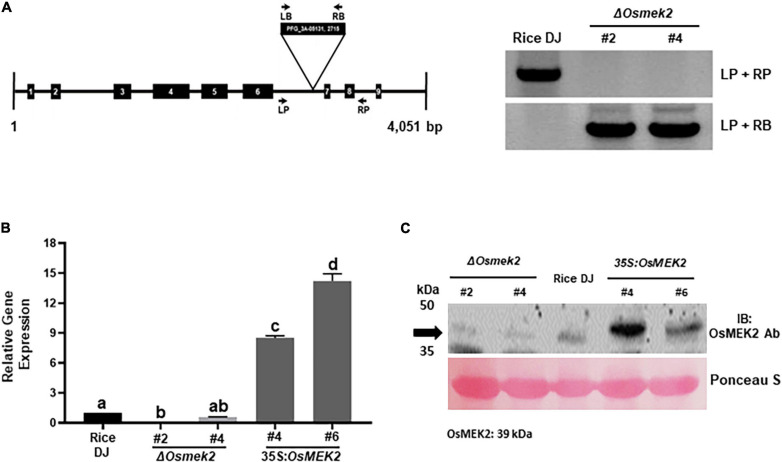
Genotyping, transcriptional, and immunoblotting analyses of Δ*Osmek2* knock-out and 35S:*OsMEK2* overexpression lines. **(A)** Genotyping of Δ*Osmek2* plants. The schematic diagram shows the T-DNA insertion site in the *OsMEK2* gene. Exons and introns are depicted by solid boxes and lines, respectively. The T-DNA insertion Δ*Osmek2* knock-out plants (M5) were detected using the gene primers (LP + RP) and the vector primers (LP + RB). LP, left primer; RP, right primer; LB, left border; RB, right border. **(B)** Transcriptional analysis of *OsMEK2* expression in wild-type (WT) rice (cultivar DJ), Δ*Osmek2* #2 and #4, and 35S:*OsMEK2* #4 and #6 plants using quantitative real-time RT-PCR. **(C)** SDS-PAGE and immunoblotting assays of *OsMEK2* expression in wild-type (WT) rice (cultivar DJ), Δ*Osmek2* #2 and #4 and 35S:*OsMEK2* #4 and #6 plants using OsMEK2 Ab (EnoGene^®^ E580135-A-SE) (∼39 kDa). Ab, antibody; IB, immunoblot; PAGE, polyacrylamide gel electrophoresis.

*OsMEK2* was knocked out in rice cultivar DJ by T-DNA insertion mutagenesis (Jeon et al., 2000). The *OsMEK2* genomic DNA sequence contains nine exons and eight introns (Supplementary Figure 4). The T-DNA insertion mutant, Δ*Osmek2*, was identified in the intronic region between the sixth and seventh exons (Figure 1A). The genotypes of Δ*Osmek2* (M5) progeny were analyzed with primer sets LP + RP and LP + LB to detect transgene and homo/hetero selection, respectively (Figure 1A). The Δ*Osmek2 #2 and*Δ*Osmek2 #4* mutants (M5) were identified as T-DNA insertion homozygous plants that lacked the specific PCR products.

*OsMEK2* expression in Δ*Osmek2* knock-out and 35S:*OsMEK2* overexpression lines was examined by quantitative real-time RT-PCR and immunoblotting (Figures 1B,C). The qRT-PCR and immunoblot analyses indicated that *OsMEK2* was not expressed in Δ*Osmek2 #2* and *#4* knock-out lines, but distinctly expressed in 35S:*OsMEK2 #4* and *#6* overexpression lines, compared to the wild-type rice DJ. These combined data indicate that the *OsMEK2* gene is knocked out in the selected Δ*Osmek2* #2 and #4 lines, but overexpressed in 35S:*OsMEK2* #4 and #6 lines. Avirulent *M. oryzae* 007 infection caused susceptible response in Δ*Osmek2* #2 and #4 lines, but resistant response in *35S:OsMEK2 #4* and *#6* overexpression lines (Supplementary Figure 5). *M. oryzae* 007 grew well and produced invasive hyphae (IH) in the leaf sheath cells of Δ*Osmek2* #2 and #4 knock-out plants, but induced hypersensitive cell death in wild-type (WT) rice DJ and 35S:*OsMEK2* #4 and #6 overexpression plants (Supplementary Figures 5A,B). The Δ*Osmek2* #2 and 35S:*OsMEK2* #4 lines were selected to use in this study, because Δ*Osmek2* #2 and #4 lines and 35S:*OsMEK2* #4 and #6 lines exhibited the same susceptible and resistant responses to *M. oryzae* 007 infection, respectively.

### The OsMEK2 Gene Is Required for Cell Death and Resistant Responses to *M. oryzae* Infection

**FIGURE 2:**
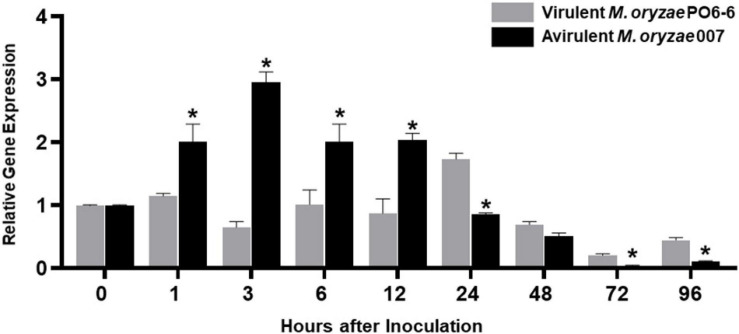
Quantitative real-time RT-PCR analysis of time-course expression of *OsMEK2* in rice leaf sheaths in the compatible and incompatible interactions of rice with *Magnaporthe oryzae*. Rice leaf sheaths were sampled at different time points after inoculation with virulent and avirulent *M. oryzae* PO6-6 and 007, respectively. *OsMEK2* expression was analyzed by quantitative RT-PCR. Relative gene expression of *OsMEK2* at each time point was calculated by normalizing with respect to expression of the internal control *OsUbiquitin* gene. Data represent the mean ± SD from three independent experiments. Asterisks above the columns indicate significant differences as analyzed by Student’s *t*-test (*P* < 0.05). hpi, hours post-inoculation.

Quantitative real-time RT-PCR analyses showed that avirulent *M. oryzae* 007 infection triggered the induction of *OsMEK2* expression at early infection times (1–12 hpi). During *M. oryzae* 007 infection, *OsMEK2* expression was up-regulated from 1 to 12 hpi and then back to the background level at 24 hpi (Figure 2). In contrast, *OsMEK2* expression by virulent *M. oryzae* PO6-6 infection remained unchanged at 1, 6, and 12 hpi, but up-regulated at 24 hpi. Notably, infection with avirulent *M. oryzae* 007 significantly reduced *OsMEK2* expression at late infection stages (72–96 hpi). These results suggest that early induction of *OsMEK2* expression is involved in rice defense signaling in the incompatible rice–*M. oryzae* interaction (Figure 2).

**FIGURE 3:**
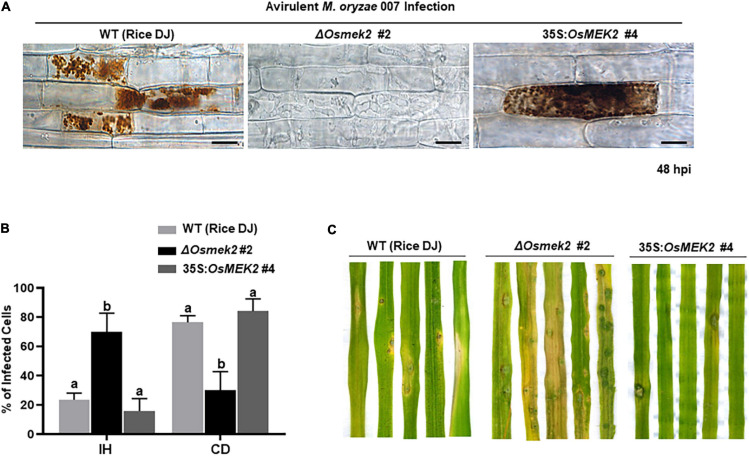
Avirulent *Magnaporthe oryzae* 007 infection causes susceptible responses in the Δ*Osmek2 #2* knock-out plants, but resistant responses in the wild-type rice and 35S:*OsMEK2* #4 overexpression plants. **(A)** Images of rice sheath epidermal cells infected with *M. oryzae* 007 (48 hpi). Rice leaf sheaths were inoculated with a conidial suspension (4 × 10^5^ conidia mL^–1^). *M. oryzae* 007 grew well and produced invasive hyphae in the *OsMEK2* knock-out (Δ*Osmek2* #2) rice, but induced hypersensitive cell death in wild-type (WT) rice cultivar DJ and *OsMEK2-*overexpressed (35S:*OsMEK2* #4) plants. Images were captured using a fluorescence microscope. hpi, hours post-inoculation. Scale bars = 20 μm. **(B)** Quantification of cell death and invasive hyphae in rice sheath cells infected with *M. oryzae* 007 (48 hpi). Results are presented as mean values ± SD; *n* = 4 leaf sheaths from different plants. Different letters above the bars indicate significantly different means (*P* < 0.05), as analyzed by Fisher’s protected least significant difference (LSD) test. IH, invasive hyphae; CD, cell death. **(C)** Disease types of rice leaves in wild-type rice (DJ), Δ*Osmek2* #2 and 35:*OsMEK2* #4 plants. Two-week-old rice seedlings were spray-inoculated with a conidial suspension (4 × 10^5^ conidia mL^–1^) of *M. oryzae* 007. Diseased leaves were photographed at 5 days after inoculation. Disease types indicate a resistant-type lesion (necrotic brownish spots) and a susceptible-type lesion (large grayish, and expanded lesions). Experiments were repeated three times with similar results.

We investigated whether *OsMEK2* is required for cell death and resistant responses to *M. oryzae* 007 infection using Δ*Osmek2* #2 and 35S:*OsMEK2* #4 rice plants (Figure 3). *M. oryzae* 007 grew poorly and caused cell death responses in leaf sheath epidermal cells of rice DJ and 35S:*OsMEK2* #4 overexpression plants at 48 hpi (Figures 3A,B). By contrast, the blast fungus grew well with plentiful invasive hyphae (IH) in the invaded Δ*Osmek2* #2 leaf sheath cells. Avirulent *M. oryzae* 007 infection induced significantly more hypersensitive cell death in rice DJ and 35S:*OsMEK2* #4 leaf sheaths than in Δ*Osmek2* #2 leaf sheaths (Figure 3B). Whole-leaf disease phenotypes were observed at 5 days after inoculation with *M. oryzae* 007 (Figure 3C). Rice DJ and 35S:*OsMEK2* #4 leaves displayed a typical resistant reaction with small necrotic, and brownish restricted lesions. By contrast, Δ*Osmek2* #2 mutant leaves displayed a typical susceptible reaction with large grayish lesions (Figure 3C). These combined results indicate that *OsMEK2* knock-out in rice DJ rendered resistance ineffective and induced susceptibility (disease) in response to avirulent *M. oryzae* infection. However, *OsMEK2* overexpression in rice DJ enhanced the cell death and resistance responses to rice blast disease.

### *OsMEK2* Knock-Out and Overexpression Differentially Regulates *MAPKs*, *WRKY* and Defense-Related Gene Expression in Rice During *M. oryzae* Infection

**FIGURE 4:**
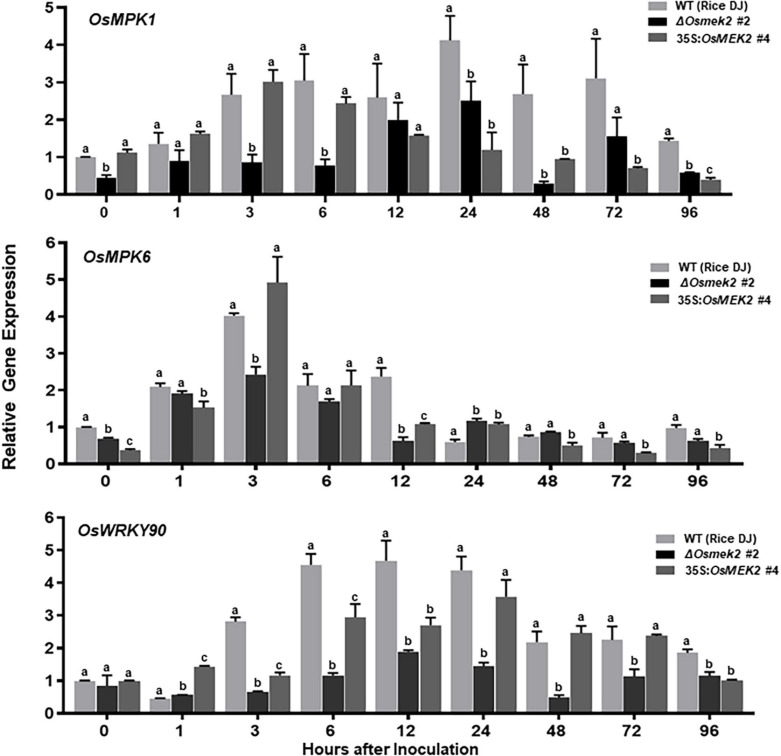
Quantitative real-time RT-PCR analysis of time-course expression of the *OsMEK2* interactors *OsMPK1*, *OsMPK6*, and *OsWRKY90* in leaf sheaths of wild-type (WT) rice (cultivar DJ), Δ*Osmek2* #2 and 35S:*OsMEK2* #4 plants infected with avirulent *Magnaporthe oryzae* 007. Leaf sheaths of wild-type (cultivar DJ), Δ*Osmek2* #2 and 35S:*OsMEK2* #4 plants were sampled at different time points after inoculation, followed by total RNA extraction. Relative gene expression of *OsMPK1*, *OsMPK6*, and *OsWRKY90* (Os09g30400) at each time point was obtained by normalizing with respect to the expression of the internal control *OsUbiquitin* (Os06g46770) gene. Data represent the means ± SD from three independent experiments. Different letters above the bars indicate significantly different means (*P* < 0.05), as analyzed by Fisher’s protected least significant difference (LSD) test.

A previous study reported that rice OsMAP2K2 (OsMEK2) interacted with and phosphorylated OsMAPKs, such as OsMPK1 and OsMPK6 (Singh et al., 2012). We analyzed *OsMPK1*, *OsMPK6*, and *OsWRKY90* expression in leaf sheaths of rice DJ, Δ*Osmek2* #2 knock-out and 35S:*OsMEK2* #4 overexpression plants during avirulent *M. oryzae* 007 infection (Figure 4). *OsMEK2* knock-out in rice DJ plants distinctly downregulated *OsMPK1* expression throughout the course of *M. oryzae* infection. However, *OsMEK2* overexpression did not upregulate expression of *OsMPK1* and *OsMPK6* in rice DJ plants. By contrast, *OsMPK6* downregulation in Δ*Osmek2* #2 leaf sheath cells was observed at early infection stages 3–12 hpi. *OsMPK6* (or *OsMPK1*) activation by *OsMKK10-2* is required for the induction of *OsWRKY45* expression and blast resistance in rice (Ueno et al., 2015). OsMPK1 is the pathogen-responsive MAPK that is involved in disease resistance (Singh et al., 2012; Ueno et al., 2015). Bimolecular fluorescence complementation (BiFC) analysis in rice leaf sheath indicates that OsMPK1 physically interacts with the OsWRKY80 transcription factor (Singh et al., 2012) and subsequently OsWRKY90 (Shen et al., 2012) as its downstream target. In plant disease resistance networks, WRKY transcription factors can associate with MAPK cascades and regulate downstream defense-related genes in the nucleus (Pandey and Somssich, 2009; Ishihama et al., 2011; Jalmi and Sinha, 2016). Avirulent *M. oryzae* 007 infection significantly upregulated *OsWRKY90* expression in rice DJ and 35S:*OsMEK2* #4 leaf sheaths, but did not affect *OsWRKY90* expression in Δ*Osmek2* #2 leaf sheaths at all tested time points after inoculation (Figure 4). This indicates that OsMEK2, the rice MAP2K, targets the OsWRKY90 transcription factor to function as a positive regulator of resistance to *M. oryzae* infection. Rice plant resistance to *M. oryzae* infection is markedly enhanced by overexpression of *OsWRKY45*, *OsWRKY53*, and *OsWRKY89* (Chujo et al., 2007; Shimono et al., 2007; Wang et al., 2007).

We next investigated the expression patterns of some defense-related genes that are induced in response to *M. oryzae* 007 infection in rice, such as pathogenesis-related protein 1b (*OsPR-1b*), phenylalanine ammonia lyase1 (*OsPAL1*), ascorbate peroxidase1 (*OsAPX1*), and *OsAPX2* (Nakashita et al., 2001; Agrawal et al., 2003; Xie et al., 2011). *OsPR-1b* expression was induced in rice DJ and 35S:*OsMEK2* #4 at all tested times, whereas it was only induced in Δ*Osmek2* #2 at 96 hpi (Supplementary Figure 6). *OsPAL1* was distinctly induced in 35S:*OsMEK2* #4 leaf sheaths during infection. *OsPAL1* expression patterns did not significantly differ in rice DJ and Δ*Osmek2* #2 leaf sheath cells at 12–96 hpi. *OsAPX1* and *OsAPX2* expression was gradually upregulated in Δ*Osmek2* #2 leaf sheath cells at 12–72 hpi (Supplementary Figure 6). These results indicate that *OsMEK2* expression positively regulates *OsPR-1b* and *OsPAL1* expression in rice during *M. oryzae* infection.

### *OsMEK2* Is Required for ROS and Ferric Ion Accumulation and Lipid Peroxidation in Rice–*M. oryzae* Interactions

**FIGURE 5:**
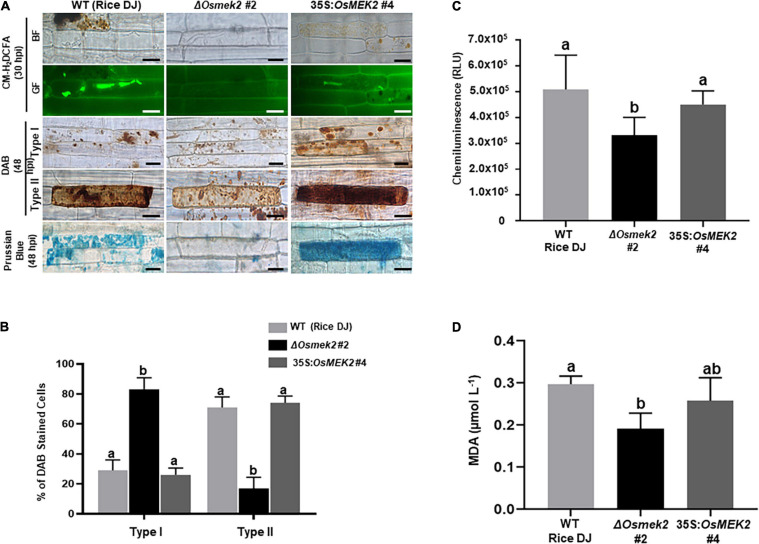
*OsMEK2* knock-out and overexpression in wild-type rice DJ differentially regulates ROS and ferric Ion (Fe^3+^) accumulation and lipid peroxidation in leaf sheaths infected with avirulent *Magnaporthe oryzae* 007. **(A)** CM-H_2_DCFDA (green fluorescence), DAB, and Prussian blue (blue color) staining shows accumulation of ROS (H_2_O_2_) and ferric ion (Fe^3+^) in rice leaf sheath epidermal cells of the wild-type (WT) rice cultivar DJ, *OsMEK2* knock-out (Δ*Osmek2* #2) and *OsMEK2-*overexpressed (35:*OsMEK2* #4) plants during *M. oryzae* infection. Scale bar = 20 μm. **(B)** Quantification of DAB-stained cells at 48 h after inoculation. The DAB-stained cells were categorized into two phenotypes: Type I, infected cells that display no or weak DAB staining; and Type II, infected cells that display strong DAB staining. Results are presented as mean values ± SD; *n* = 4 leaf sheaths from different plants. **(C)** Quantification of ROS production in rice leaf sheaths at 48 h after inoculation. ROS production was quantified by a luminol-based assay using a GloMax^®^ 96 Microplate Luminometer (Promega). Values are means ± SD of total relative luminescent units (RLU) (*n* = 10). **(D)** Lipid (MDA) peroxidation determination in rice leaf sheaths at 48 h after inoculation. Results are presented as mean values ± SD; *n* = 4 leaf sheaths from different plants. Images were captured using a fluorescence microscope (Zeiss equipped with Axioplan 2) with bright field and a combination of excitation (450–490 nm) and emission (515–565 nm) GF filters. Experiments were repeated three times with similar results. Different letters above the bars indicate significantly different means (*P* < 0.05), as analyzed by Fisher’s protected least significant difference (LSD) test. BF, bright field; GF, green fluorescence; hpi, hours post-inoculation; MDA, malondialdehyde.

We analyzed ROS and ferric ion (Fe^3+^) accumulation and lipid [malondialdehyde (MDA)] peroxidation in leaf sheath cells of rice DJ, 35S:*OsMEK2* #4 and Δ*Osmek2* #2 plants during avirulent *M. oryzae* 007 infection to determine whether *OsMEK2* is involved in iron- and ROS-dependent ferroptotic cell death (Figure 5). CM-H_2_DCFDA (green fluorescence) and DAB (dark brown) staining revealed that ROS (H_2_O_2_) strongly accumulated inside and around invasive hyphae (IH) in rice DJ and 35S:*OsMEK2* #4 epidermal cells at 30–48 hpi (Figure 5A). By contrast, ROS did not accumulate around invasive hyphae (IH) in Δ*Osmek2* #2 epidermal cells after avirulent *M. oryzae* 007 infection. The ROS-sensitive CM-H_2_DCFDA dye is an indicator that can be used to monitor ROS localization in living plant cells (Kristiansen et al., 2009). CM-H_2_DCFDA-specific ROS-localized fluorescence was clearly visible around invasive hyphae (IH) and cellular membranes in rice DJ and 35S:*OsMEK2* #4 cells, whereas ROS-localized fluorescence was absent or weakly visible around invasive hyphae (IH) in Δ*Osmek2* #2 cells at 30 hpi (Figure 5A). DAB is oxidized by H_2_O_2_ in the presence of peroxidase to generate a dark brown precipitate, which indicates the presence and distribution of H_2_O_2_ in plant cells (Fryer et al., 2002; Kristiansen et al., 2009). We classified DAB-stained cells into two phenotypes: Type I infected cells display no or weak DAB staining, and Type II infected cells display strong DAB staining (Figure 5B). Most of the infected cells displayed strong brown staining (Type II phenotype) in rice DJ and 35S:*OsMEK2* #4 cells. By contrast, significantly fewer Δ*Osmek2* #2 cells displayed DAB staining at 48 hpi. A chemiluminescent assay with a luminometer revealed that ROS levels were significantly lower in Δ*Osmek2* #2 cells than in DJ and 35S:*OsMEK2* #4 cells at 48 hpi (Figures 5A–C).

Ferric ion (Fe^3+^) accumulation and localization in rice cells was detected by Prussian blue (blue color) staining of rice leaf sheath cells at 48 hpi with avirulent *M. oryzae* 007 (Figure 5A). Rice DJ and 35S:*OsMEK2* #4 epidermal cells displayed strong blue staining, whereas Δ*Osmek2* #2 epidermal cells did not display blue stain. Next, we analyzed oxidative damage and lipid (MDA) peroxidation in rice leaf sheath cells at 48 hpi with *M. oryzae* 007 (Figure 5D) by performing the MDA assay as described previously (Zhang et al., 2009; Dangol et al., 2019). Lipid peroxidation levels were significantly lower in Δ*Osmek2* #2 cells than in rice DJ. The MDA level in 35S:*OsMEK2* #4 was similar to that in rice DJ. These combined results indicate that OsMEK2 has crucial roles in ROS and Fe^3+^ accumulation and lipid peroxidation during the ferroptotic cell death response in rice.

### Erastin Triggers Iron- and ROS-Dependent Ferroptotic Cell Death in Δ*Osmek2* Knock-Out Mutant Plants During *M. oryzae* Infection

**FIGURE 6:**
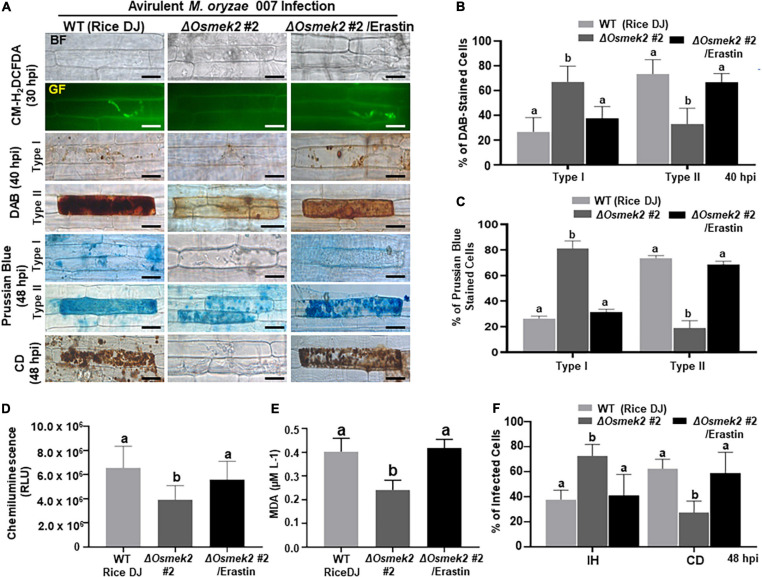
The small molecule inducer erastin triggers iron- and ROS-dependent ferroptotic cell death in the compatible Δ*Osmek2*–*Magnaporthe oryzae* interaction. Leaf sheaths of WT rice DJ and Δ*Osmek2* #2 knock-out plants were inoculated with conidial suspensions (4 × 10^5^ conidia/mL) of *M. oryzae* 007 containing 10 μM erastin. **(A)** Erastin treatment recovered ROS and ferric ion (Fe^3+^) accumulation and cell death in leaf sheaths of Δ*Osmek2* #2 plants during avirulent *M. oryzae* 007 infection. ROS accumulation in leaf sheath epidermal cells was detected by CM-H_2_DCFDA (green fluorescence) and DAB (dark brown color) staining. Prussian blue (blue color) staining shows ferric ion accumulation in rice cells. The images are representative of different leaf sheath samples from three independent experiments. Scale bars = 20 μm. **(B)** DAB-stained cell phenotypes. DAB-stained cells were divided into two phenotypes: Type I, cells that contain invasive hyphae (IH) but are weakly or not DAB-stained; and Type II, strongly DAB-stained cells with only a few poor hyphae. **(C)** Quantification of Prussian blue-stained cells. Prussian blue-stained cells were divided into two phenotypes: Type I, cells that contain invasive hyphae (IH) but are weakly or not Prussian blue-stained; and Type II, strongly Prussian blue-stained with only a few poor hyphae. **(D)** Quantification of ROS accumulation. ROS accumulation was monitored using a GloMax^®^ 96 Microplate Luminometer (Promega). Values are means ± SD of total relative luminescent units (RLU) (*n* = 10). **(E)** Determination of lipid (MDA) peroxidation in leaf sheaths at 48 h after inoculation. Results are presented as means ± SD; *n* = 4 leaf sheaths from different plants. **(F)** Quantification of infected cell phenotypes in rice leaf sheaths. Images were taken using a fluorescence microscope (Zeiss equipped with Axioplan 2) with bright field and green fluorescence (GF) filters. Experiments were repeated three times with similar results. Results are presented as means ± SD; *n* = 4 leaf sheaths. Different letters above the bars indicate significantly different means (*P* < 0.05), as analyzed by Fisher’s protected least significant difference (LSD) test. IH, invasive hyphae; CD, cell death; BF, bright field; GF, green fluorescence; hpi, hours post-inoculation.

Erastin is a small molecule inducer that triggers ferroptotic cell death in mammals and plants (Dixon et al., 2012; Dangol et al., 2019). Treatment with 10 μM erastin triggered ROS (H_2_O_2_) and Fe^3+^ accumulation and cell death response in Δ*Osmek2* #2 leaf sheaths during avirulent *M. oryzae* 007 infection (Figure 6). However, mock (water) or 10 μM erastin treatment did not trigger ROS (H_2_O_2_) and Fe^3+^ accumulation in healthy rice DJ leaf sheaths (Supplementary Figure 7). CM-H_2_DCFDA and DAB staining detected H_2_O_2_ accumulation in Δ*Osmek2* #2 leaf sheath cells at 30∼48 hpi with *M. oryzae* conidial suspension containing 10 μM erastin (Figures 6A,B). Erastin treatment during *M. oryzae* 007 infection induced H_2_O_2_ accumulation in Δ*Osmek2* #2 cells at 48 hpi as detected with a luminometer (Figure 6D). Erastin induced Fe^3+^ accumulation and increased the number of Prussian blue-stained cells in Δ*Osmek2* #2 leaf sheaths at 48 hpi (Figures 6A,C). Iron-dependent MDA peroxidation was upregulated at 48 hpi in Δ*Osmek2* #2 leaf sheath cells by treating with erastin (Figure 6E). Erastin treatment significantly enhanced the cell death response in Δ*Osmek2* #2 cells during *M. oryzae* infection (Figures 6A,F). These combined results indicate that erastin triggers iron- and lipid ROS-dependent, but *OsMEK2*-independent, ferroptotic cell death in rice during *M. oryzae* infection.

### Disease-Related Cell Death Is ROS-Dependent but Iron-Independent in Δ*Osmek2* Knock-Out Mutant Plants During the Late Stage of *M. oryzae* Infection

**FIGURE 7:**
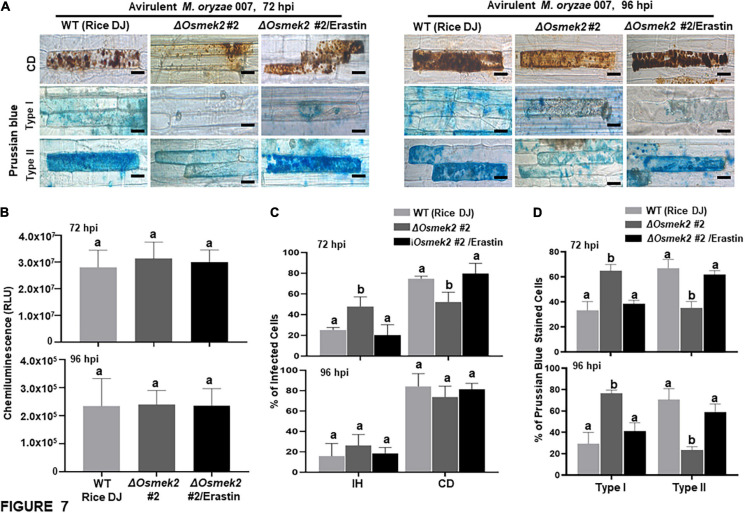
Disease-related cell death is ROS-dependent and iron-independent in the compatible Δ*Osmek2*–*Magnaporthe oryzae* interaction. Δ*Osmek2* #2 leaf sheaths were inoculated with conidial suspensions (4 × 10^5^ conidia mL^–1^) of *M. oryzae* 007 containing 10 μM erastin. *M. oryzae* 007 infection induced disease-related cell death, but Δ*Osmek2* #2 leaf sheath cells did not accumulate ferric ions (Fe^3+^) at 96 h after inoculation. **(A)** Erastin treatment induces cell death and ferric ion (Fe^3+^) accumulation in Δ*Osmek2* #2 leaf sheath cells during avirulent *M. oryzae* 007 infection. Prussian blue (blue color) staining shows ferric ion accumulation in rice cells. The images are representative of different leaf sheath samples from three independent experiments. Scale bars = 20 μm. **(B)** Quantification of ROS accumulation in leaf sheath cells. ROS quantities were monitored using a GloMax^®^ 96 Microplate Luminometer (Promega). Values are means ± SD of total relative luminescent units (RLU) (*n* = 10). **(C)** Quantification of infected cell phenotypes in rice leaf sheaths. Results are presented as mean values ± SD; *n* = 4 leaf sheaths from different plants. **(D)** Quantification of Prussian blue-stained cells. Prussian blue-stained cells were categorized into two phenotypes: Type I, cells that contain invasive hyphae (IH) but are weakly or not Prussian blue-stained; and Type II, strongly Prussian blue-stained with only a few poor hyphae. Images were taken using a fluorescence microscope (Zeiss equipped with Axioplan 2). Experiments were repeated three times with similar results. Results are presented as means ± SD; *n* = 4 leaf sheaths. Different letters above the bars indicate significantly different means (*P* < 0.05), as analyzed by Fisher’s protected LSD test. IH, invasive hyphae; CD, cell death; BF, bright field; GF, green fluorescence; hpi, hours post-inoculation.

**FIGURE 8:**
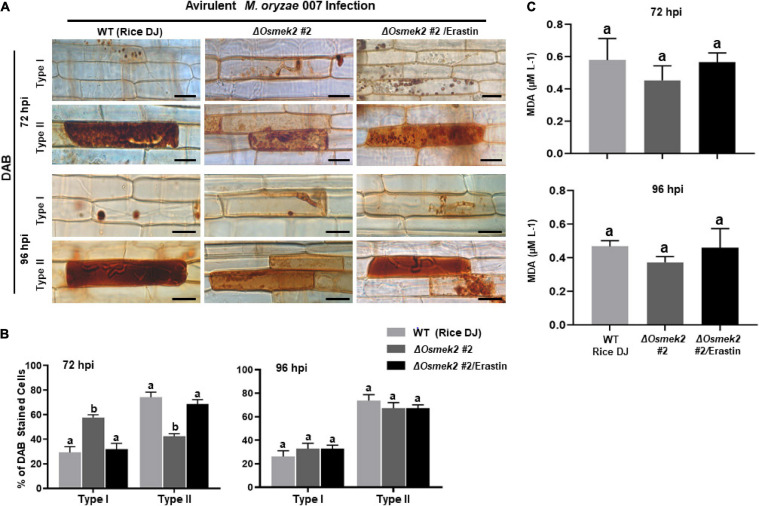
ROS accumulation and lipid peroxidation in leaf sheaths of wild-type (WT) rice (cultivar DJ) and Δ*Osmek2* #2 plants at 72 and 96 h after inoculation with avirulent *Magnaporthe oryzae* 007 with 10 μM erastin. Rice Δ*Osmek2* #2 leaf sheaths were inoculated with conidial suspensions (4 × 10^5^ conidia mL^–1^) of avirulent *M. oryzae* 007 with and without 10 μM erastin. Avirulent *M. oryzae* 007 infection induced ROS production and lipid peroxidation in leaf sheaths of the susceptible Δ*Osmek2* plants at 96 hpi, which was similar to those in erastin-treated leaf sheaths of the susceptible Δ*Osmek2* #2 plants. **(A)** DAB-stained cell phenotypes at 72 and 96 h after inoculation. DAB-stained cells were categorized into two phenotypes: Type I, cells that contain invasive hyphae (IH) but are weakly or not DAB-stained; and Type II, strongly DAB-stained cells with only a few poor hyphae. Scale bars = 20 μm. **(B)** Quantification of DAB-stained cells at 72 and 96 h after inoculation. DAB-stained cells were categorized into two phenotypes: Type I, infected cells that display no or weak DAB staining; Type II, infected cells that display strong DAB staining. **(C)** Determination of lipid (MDA) peroxidation in rice leaf sheaths at 72 and 96 h after inoculation. Results are presented as mean values ± SD; *n* = 4 leaf sheaths from different plants. Images were captured using a fluorescence microscope (Zeiss equipped with Axioplan 2). Results are presented as mean values ± SD; *n* = 4 leaf sheaths from different plants. Different letters above the bars indicate significantly different means (*P* < 0.05) as analyzed by Fisher’s protected LSD test. Experiments were repeated three times with similar results. hpi, hours post-inoculation.

Reactive oxygen species (H_2_O_2_) and Fe^3+^ did not accumulate in healthy rice DJ leaf sheaths at 72 and 92 h after treatment with 10 μM erastin (Supplementary Figure 7). Erastin treatment strongly induced HR cell death, ROS and Fe^3+^ accumulation, and lipid peroxidation in Δ*Osmek2* #2 leaf sheaths at 72 and 96 hpi with avirulent *M. oryzae* 007, similar to that observed in rice DJ (Figures 7, 8). By contrast, *M. oryzae* 007 infection induced disease-related cell death but not Fe^3+^ accumulation in erastin-untreated leaf sheaths of the susceptible Δ*Osmek2* #2 cells at 72 and 96 hpi (Figures 7A,C,D). However, the chemiluminescent assay indicated that the high ROS levels observed in the Δ*Osmek2* #2 cells were similar to those observed in rice DJ and erastin-treated Δ*Osmek2* #2 leaf sheaths at 72 and 96 hpi (Figure 7B). ROS accumulation, MDA peroxidation, and cell death phenotypes were distinctly enhanced in the Δ*Osmek2* #2 cells (Figures 7A,C, 8A–C); however, increased Fe^3+^ accumulation was not observed at 72 and 96 hpi (Figures 7A,D). Avirulent *M. oryzae* 007 infection did not increase the number of Prussian blue-stained cells in Δ*Osmek2* #2 leaf sheaths at 72 and 96 hpi (Figure 7D). These combined results indicate that disease-related cell death is ROS-dependent but iron-independent in the compatible rice–*M. oryzae* interaction. Increased ROS production and lipid peroxidation in *M. oryzae*-infected tissues may induce susceptibility-related cell death that facilitates subsequent fungal invasion and infection. However, intracellular iron accumulation may not be required for disease-related cell death in compatible rice–*M. oryzae* interactions.

### *OsMPK1* Overexpression Induces Iron- and ROS-Dependent Ferroptotic Cell Death in Rice During *M. oryzae* Infection

**FIGURE 9:**
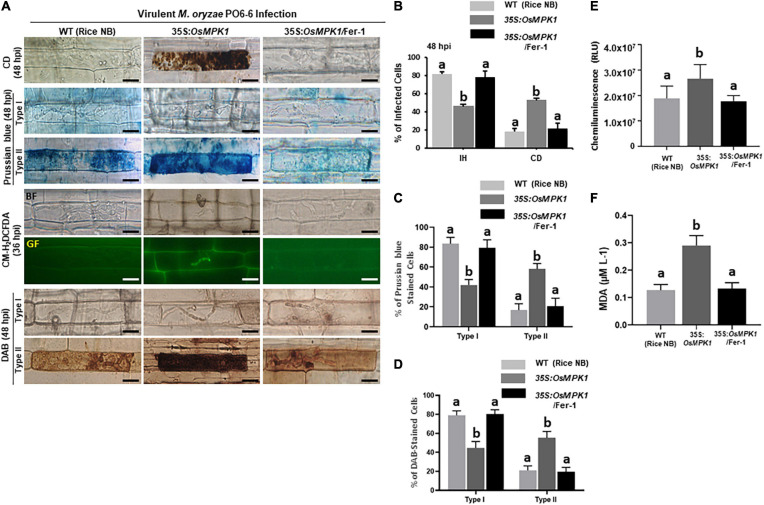
*OsMPK1* overexpression induces ROS and ferric Ion (Fe^3+^) accumulation, lipid peroxidation and cell death in rice leaf sheaths during virulent *Magnaporthe oryzae* PO6-6 infection. Leaf sheaths of the susceptible wild-type (WT) cultivar NB and *OsMPK1*-overexpressed (35S:*OsMPK1*) plants were inoculated with the conidial suspension (4 × 10^5^ conidia/mL) of virulent *M. oryzae* PO6-6 and then treated with 10 μM Fer-1. **(A)** Microscopic images of cell death and ROS and ferric Ion (Fe^3+^) accumulation in rice sheath cells untreated or treated with 10 μM Fer-1 at 48 hpi. ROS accumulation in the infected leaf sheath epidermal cells was detected by CM-H_2_DCFDA (green fluorescence) and DAB (dark brown color) staining. Prussian blue (blue color) staining shows ferric ion accumulation in rice cells. The images are representatives of different leaf sheath samples from three independent experiments. Scale bars = 20 μm. **(B)** Quantification of cell death (CD) and invasive hyphae (IH) in rice sheath cells at 48 hpi. **(C)** Quantification of Prussian blue-stained cells. Prussian blue-stained cells were divided into two phenotypes: Type I, cells that contain invasive hyphae (IH) but are weakly or not Prussian blue-stained; and Type II, strongly Prussian blue-stained with only a few poor hyphae. **(D)** DAB-stained cell phenotypes. DAB-stained cells were divided into two phenotypes: Type I, cells that contain invasive hyphae (IH) but are weakly or not DAB-stained; and Type II, strongly DAB-stained cells with only a few poor hyphae. **(E)** Quantification of ROS accumulation. ROS accumulation was monitored using a GloMax^®^ 96 Microplate Luminometer (Promega). Values are means ± SD of total relative luminescent units (RLU) (*n* = 10). **(F)** Determination of lipid peroxidation by MDA (malondialdehyde) assay. Images were taken using a fluorescence microscope (Zeiss equipped with Axioplan 2) with bright field and green fluorescence (GF) filters. Experiments were repeated three times with similar results. Results are presented as mean values ± SD; *n* = 4 leaf sheaths from different plants. Different letters above the bars indicate significantly different means (*P* < 0.05) as analyzed by Fisher’s protected LSD test. Fer-1, ferrostatin-1, hpi, hours post-inoculation.

Rice MAP kinase (OsMPK1) is an interactor of OsMEK2 and actively involved in *M. oryzae* infection (Singh et al., 2012; Ueno et al., 2015). Genomic DNA sequence of OsMPK1 contains six exons and five introns (Supplementary Figure 8). Amino acid sequence alignments of OsMPK1 with other plant MPKs indicated that OsMPK1 shares 67.95–96.20% homology with MAPKs of rice, Arabidopsis, tomato, and maize (Supplementary Figure 9). In particular, OsMPK1 has high levels of identity with OsMPK6 (96.20%) and AtMPK6 (83.76%) (Supplementary Figure 9). OsMPK1 was also phylogenetically close to OsMPK6 and AtMPK6 (Supplementary Figure 10). We overexpressed *OsMPK1* in the susceptible rice cultivar Nipponbarre (NB) under the control of CaMV 35S promoter. *OsMPK1* was distinctly overexpressed in leaf sheath cells of 35S:*OsMPK1-*transformed plants (Supplementary Figure 11). 35S:*OsMPK1* overexpression induced a hypersensitive cell death with poorly grown invasive hyphae (IH) in leaf epidermal cells at 48 hpi (Figures 9A,B). DAB and CM-H_2_DCFDA staining showed accumulation of ROS around the IH in 35S:*OsMPK1* leaf sheath cells at 36–48 hpi during virulent *M. oryzae* PO6-6 infection (Figures 9A,D). Chemiluminescence assay with a luminometer revealed that ROS levels distinctly increased in 35S:*OsMPK1* overexpression cells at 48 hpi (Figure 9E). Prussian blue staining of Fe^3+^ showed strong accumulation of ferric ion in 35S:*OsMPK1* cells at 48 hpi (Figures 9A,C). Lipid (MDA) peroxidation levels were significantly higher in 35S:*OsMPK1* overexpression cells than in rice NB cells at 48 hpi (Figure 9F). By contrast, ferostatin-1(Fer-1) treatments distinctly inhibited iron- and ROS-dependent ferroptotic cell death during infection, which ultimately led to the restored normal hyphal growth in 35S:*OsMPK1* overexpression cells (Figure 9). ROS and ferric ion accumulation and lipid peroxidation nearly disappeared in Fer-1-treated 35S:*OsMPK1* leaf sheaths during infection (Figures 9A,B,D–F). The combined results indicate that *OsMPK1* is involved in ROS and Fe^3+^ accumulation and lipid peroxidation leading to the ferroptotic cell death during *M. oryzae* infection.

### *OsMEK2* Expression Positively Regulates *OsNADP-ME* and *OsRbohB* Expression in Rice During *M. oryzae* 007 Infection

**FIGURE 10:**
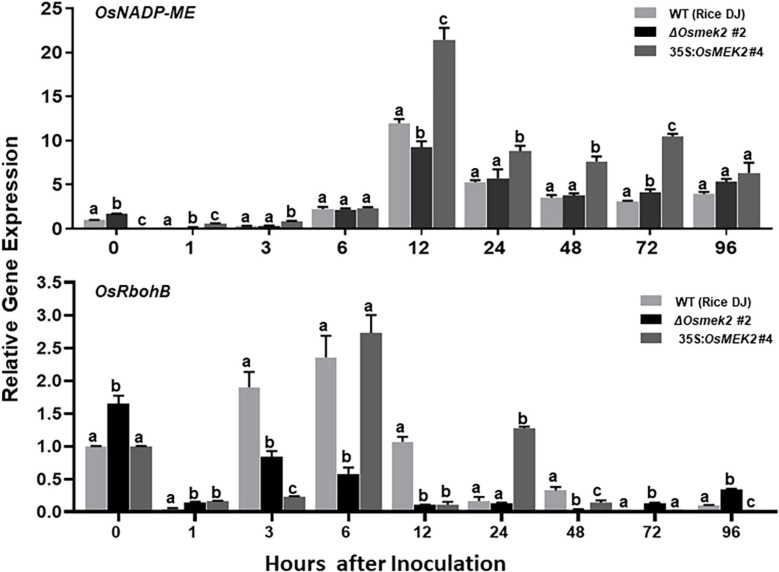
Quantitative real-time RT-PCR analysis of time-course expression of *OsNADP-ME* and *OsRbohB* in rice leaf sheaths infected with avirulent *Magnaporthe oryzae* 007. Leaf sheaths of the wild-type (WT) rice cultivar DJ, *OsMEK2* knock-out (Δ*Osmek2* #2) and *OsMEK2-*overexpressed (35:*OsMEK2* #4) plants were sampled at different time points after inoculation, followed by total RNA extraction. Relative gene expression levels of *OsNADP-ME* (Os01g52500) and *OsRbohB* (Os01g25820) at each time point were calculated by normalizing with respect to the expression of the internal control *18S rRNA* (XR_003238819.1) gene. Data represent the means ± SD from three independent experiments. Different letters above the bars indicate significantly different means (*P* < 0.05), as analyzed by Fisher’s protected LSD test.

We recently reported that rice NADP-malic enzyme (OsNADP-ME) and respiratory burst oxidase homolog (OsRboh, NADPH-oxidase) are involved in Fe^3+^ and ROS accumulation during cell death and defense responses in rice (Dangol et al., 2019). Interaction of *N. benthamiana* WRKY8 with MAPKs induce the downstream target genes *NADP-ME* and *Rboh*, resulting in the ROS burst (Yoshioka et al., 2003; Ishihama et al., 2011). RbohB activation via MAPK cascades is required for the pathogen-responsive ROS burst (Adachi and Yoshioka, 2015). Here, we analyzed the expression of *OsNADP-ME2-3* (Singh et al., 2016) and *OsRbohB* (Wong et al., 2007) in wild-type rice DJ, Δ*Osmek2* #2 knock-out and 35S:*OsMEK2* #4 overexpression plants during avirulent *M. oryzae* 007 infection (Figure 10). *OsNADP-ME2-3* expression patterns did not differ in rice DJ and Δ*Osmek2* #2 plants, except for a reduction in Δ*Osmek2* #2 plants at 12 hpi. However, *OsNADP-ME2-3* was distinctly expressed in 35S:*OsMEK2* #4 plants at 12 and 72 hpi. *OsRbohB* expression was significantly downregulated in Δ*Osmek2* #2 plants, but distinctly upregulated at 96 hpi, compared to that in rice DJ (Figure 10). These combined results indicate that *OsMEK2* expression positively regulates *OsNADP-ME* and *OsRbohB* expression during avirulent *M. oryzae* infection.

### Subcellular Localization of OsMEK2, OsMPK1, and OsWRKY90

**FIGURE 11:**
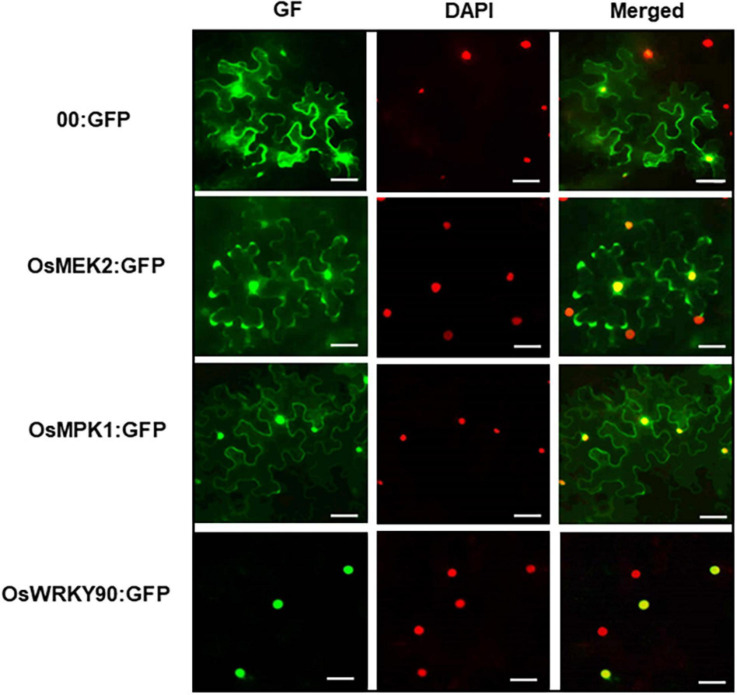
Subcellular localization of OsMEK2, OsMPK1, and OsWRKY90 at 36 h after agroinfiltration into *Nicotiana benthamiana* leaves. 4′,6-diamidino-2-phenylindole (DAPI) staining was used to visualize nuclei in *N. benthamiana* epidermal cells. Images of subcellular localization of 00:GFP, OsMEK2:GFP, OsMPK1:GFP, and OsWRKY90:GFP were taken with a fluorescence microscope using bright field, GF (green fluorescence) and DAPI filters. GFP, green fluorescent protein. Scale bars = 50 μm.

The subcellular localization study of MAP kinase signaling proteins is important for understanding their biological functions in plant cells. In this study, we investigated subcellular localization of green fluorescent protein (GFP)-tagged 35S:OsMEK2 (OsMEK2:GFP), OsMPK1:GFP, and OsWRKY90:GFP in *N. benthamiana* leaves using *A. tumefaciens*-mediated transient expression (Figure 11). The nuclei inside cells were counterstained with DAPI to help verify nuclear localization of GFP-tagged proteins. The control GFP construct (00:GFP) was ubiquitously detected in the cytoplasm of *N. benthamiana* cells. OsMEK2:GFP was localized mainly to the cytoplasm, but also to some nuclei in *N. benthamiana* cells. OsMPK1:GFP was localized to both the cytoplasm and nuclei. However, the OsWRKY90:GFP transcription factor was located inside the nuclei, but not in the cytoplasm. These results indicate that OsMEK2 interacts with OsMPK1 in the cytoplasm, and OsMPK1 moves into the nuclei to interact with the OsWRK90 transcription factor.

## Discussion

Plant mitogen-activated protein kinase (MAPK) cascades are involved in signaling multiple defense responses, the HR, and cell death responses during pathogen invasion and infection (Meng and Zhang, 2013; Thulasi Devendrakumar et al., 2018). We recently reported a ferroptotic cell death response in rice (*Oryza sativa*) during *Magnaporthe oryzae* infection (Dangol et al., 2019). Ferroptosis is a form of non-apoptotic iron-dependent cell death that was first discovered in oncogenic mammalian cells (Dixon et al., 2012). OsMEK2 interacts with OsMPK1 (Singh et al., 2012). Here, we demonstrated that rice MAP kinase (OsMEK2 and OsMPK1) signaling was required for iron- and ROS-dependent ferroptotic cell death in rice–*M. oryzae* interactions, and blast disease (susceptibility)-related cell death was ROS-dependent but iron-independent in the susceptible Δ*Osmek2* mutant plants.

We previously reported that OsMEK2 physically interacts with and phosphorylates downstream OsMPK1 and OsMPK6 (Singh et al., 2012). MAPK kinase (MEK)–MAPK interactions may have functional roles in HR cell death responses and MAPK signaling networks during *M. oryzae* infection in rice plants. In the present study, *OsMEK2* knock-out in rice DJ plants induced a susceptible (disease) response to *M. oryzae* infection in Δ*Osmek2* knock-out plants; however, *OsMEK2* overexpression in 35S:*OsMEK2* plants redeemed hypersensitive cell death response against *M. oryzae* infection. These results suggest the HR-mediated resistance signaling of OsMEK2 during *M. oryzae* infection. *OsMEK2* knock-out and overexpression differentially regulated *OsMPK1*, *OsMPK6*, and *OsWRKY90* expression in Δ*Osmek2* and 35S:*OsMEK2* plants, especially during early stages of *M. oryzae* infection. These results indicated that *OsMEK2* expression distinctly induced the downstream *OsMPK1* and *OsMPK6* signaling responses to *M. oryzae* infection. OsMPK1 physically interacts with the OsWRKY80 (Singh et al., 2012) and OsWRKY90 (Shen et al., 2012) transcription factors. In plant disease resistance networks, WRKY transcription factors can associate with MAP kinases in the nuclei and regulate downstream defense-related gene expression (Pandey and Somssich, 2009; Ishihama et al., 2011; Jalmi and Sinha, 2016). *OsMEK2* expression distinctly induced pathogenesis-related protein 1b (*OsPR-1b*) but not ascorbate peroxidase1/2 (*OsAPX1/2*) during *M. oryzae* infection. PR-1 proteins are markers of defense responses to pathogen infection in rice (Mitsuhara et al., 2008). Thus, *OsMEK2* signaling may trigger the MAP kinase cascade pathways leading to HR-mediated resistance to *M. oryzae* infection.

Mitogen-activated protein kinase signaling cascades are highly conserved in diverse plant species and involved in plant defense responses (Tanaka et al., 2009; Melech-Bonfil and Sessa, 2011; Oh et al., 2013; Ma et al., 2017). Activation of the MAPKK-MAPK cascades is associated with programmed cell death (PCD) in plants (Thulasi Devendrakumar et al., 2018). ROS mediate cellular defense responses against pathogen invasion in plants (Apel and Hirt, 2004; Mittler et al., 2004). Ferric ion (Fe^3+^) is essential in plants for HR cell death and defense- and disease-related iron homeostasis (Liu et al., 2007; Dangol et al., 2019). Iron is required for intracellular lipid peroxide accumulation (Stockwell et al., 2017; Dangol et al., 2019). Pathogen-responsive MAPKs may trigger the early ROS burst during plant defense and cell death responses (Meng and Zhang, 2013). Our study suggests that *OsMEK2* activation is one of the earliest signaling events involved in iron- and ROS-dependent ferroptotic cell death in rice. Iron and ROS accumulation was not induced in Δ*Osmek2* knock-out leaf sheaths during early *M. oryzae* infection. The ROS burst in rice may originate from the plasma membrane NADPH-oxidase (*OsRbohB*), which is activated during early *M. oryzae* infection. MAPKs could phosphorylate WRKY transcription factors to subsequently activate NADPH oxidases (Rbohs), which are essential for potent and prolonged ROS burst (Adachi et al., 2015; Jwa and Hwang, 2017). Early MAPK kinase (*OsMEK2*) signaling seems likely to activate *OsMPK1*, *OsWRKY90*, and NADPH-oxidase (*OsRbohB*), ultimately leading to the iron- and ROS-dependent ferroptotic cell death response. In our study, overexpression of 35S:*OsMPK1* significantly induced iron and ROS accumulation during infection. However, the ferroptosis inhibitor, ferrostatin-1 (Fer-1), suppressed iron- and ROS-dependent ferroptotic cell death, which ultimately led to the restored normal invasive hyphal growth in 35S:*OsMPK1* overexpression plants. Ma et al. (2017) demonstrated that overexpression of rice *MPK6* (*OsMPK1* in current study) reduced susceptibility in rice cultivar Zhonghua 11 against *Xanthomonas oryzae* pv. *oryzae* infection. Our results suggest that the OsMEK2-OsMPK1-OsWRKY90 cascades positively regulate ferroptotic cell death in rice against *M. oryzae* infection. Rice MPKK10.2 and MPK6 cascades induced resistance against *X. oryzae pv. oryzae* infection (Ma et al., 2017). Plant MAP kinases have been demonstrated to differentially regulate WRKY transcription factors in defense-related signaling pathways (Eulgem and Somssich, 2007; Pandey and Somssich, 2009; Ishihama and Yoshioka, 2012). Jalmi and Sinha (2016) reported positive involvement of the OsMKK3-OsMPK7-OsWRKY30 module in inducing rice resistance against *X. oryzae* infection. Rice OsMPK7 interacts with and phosphorylates OsWRKY30 to mediate resistance against *X. oryzae* infection (Jalmi and Sinha, 2016). Erastin is an oncogenic RAS-selective lethal (RSL) small molecule that effectively damages human cancer cells but does not affect isogenic normal cells (Dolma et al., 2003). Dixon et al. (2012) first discovered that erastin induces cellular iron-dependent lipid ROS accumulation in mammalian cells, leading to the unique iron-dependent non-apoptotic cell death (ferroptosis). The ferroptosis inducer erastin inhibits glutathione peroxidase 4 (GPX4) activity to elevate cytoplasmic lipid ROS levels (Yang and Stockwell, 2016; Stockwell et al., 2017). GPX4 is an inhibitor of lipid peroxidation (Ursini et al., 1982), and reduces membrane phospholipid hydroperoxides to suppress ferroptosis (Stockwell et al., 2017). In this study, we showed that erastin treatment of Δ*Osmek2* knock-out mutant rice triggered iron and ROS accumulation and lipid peroxidation, leading to iron- and lipid ROS-dependent ferroptotic cell death during *M. oryzae* infection. The erastin-induced ferroptotic cell death in rice is iron- and lipid ROS-dependent, but is independent of the rice MAPK kinase OsMEK2. In our earlier study, we validated these results by showing that erastin treatment triggered *OsMADP-ME2*–independent ferroptotic cell death in rice (Dangol et al., 2019). NADP-ME provides the cytoplasmic electron donor NADPH for ROS production (Singh et al., 2016; Jwa and Hwang, 2017). Thus, erastin-mediated induction of ferroptotic cell death in rice may not require specific cell death-related plant genes, such as *OsMEK2* and *OsNADP-ME2*. Plant and mammalian genes that are specifically regulated by erastin to trigger ferroptotic cell death have not yet been identified (Stockwell et al., 2017; Hirschhorn and Stockwell, 2019). Erastin-induced ferroptotic cell death in rice does not appear to be genetically controlled, but may occur non-specifically.

Disease-related cell death occurs during compatible (susceptible) interactions between plants and pathogens (Greenberg, 1997; Richberg et al., 1998). In this study, we showed that *M. oryzae* infection induced disease-related cell death that was not dependent on iron accumulation in rice cells at the late infection stage. ROS accumulation, lipid peroxidation, and cell death phenotypes distinctly increased in the Δ*Osmek2* knock-out at the late *M. oryzae* infection stages (72 and 96 hpi); however, significant iron accumulation did not occur. Iron-independent and ROS-dependent cell death at late infection stages in the compatible rice–*M. oryzae* interaction is distinct from ferroptotic HR cell death, but similar to necrosis-like cell death. Necrotic cell death caused by compatible plant interactions with necrotrophic pathogens is dependent on ROS accumulation (Mengiste, 2012). A toxin or secreted virulence factor from the microbial pathogen may directly kill plant cells or trigger an endogenous cell death program (Greenberg, 1997). Cell death in compatible interactions may derive from pathogen-mediated necrosis rather than host-induced PCD (Morel and Dangl, 1997; Gilchrist, 1998). ROS accumulation and lipid peroxidation during *M. oryzae* infection at the late stages are involved in disease (susceptibility)-related cell death in rice, as suggested previously (Govrin and Levine, 2000; Greenberg and Yao, 2004; Torres et al., 2006; Choi et al., 2013; Jwa and Hwang, 2017). However, intracellular iron accumulation may not be required for disease-related cell death in compatible rice–*M. oryzae* interactions. By contrast, iron accumulation is likely essential for the induction of ferroptotic cell death to restrict avirulent *M. oryzae* invasion into rice cells.

**FIGURE 12:**
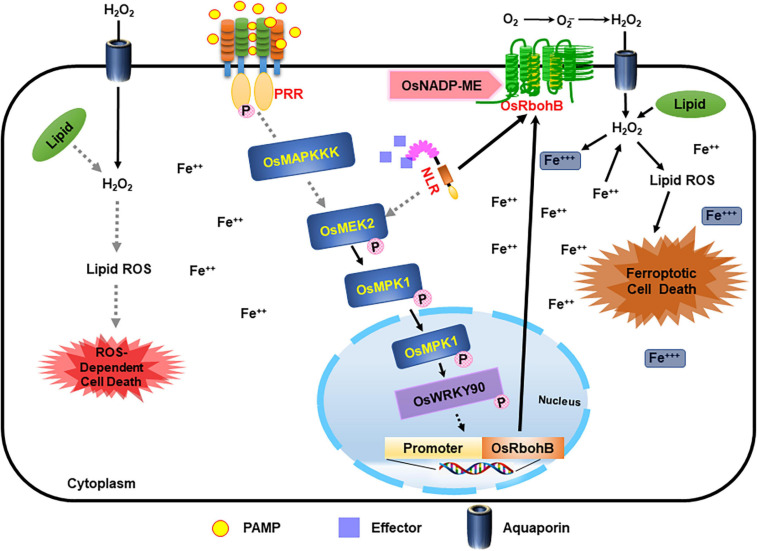
Proposed model of rice MAP kinase kinase 2 (OsMEK2) signaling pathways leading to iron- and ROS-dependent ferroptotic cell death in incompatible rice–*Magnaporthe oryzae* interactions. *M. oryzae* invasion in rice cells activates rice MAP kinase kinase 2 (OsMEK2) via different hypothesized signaling pathways (gray dotted lines). The perception of PAMPs or pathogen effectors via membrane-bound PRRs or NLRs, respectively, activates MAP kinases in plant cells. Active OsMEK2 triggers OsMPK1-OsWRKY90 pathways in the nucleus, which may lead to upregulation of NADP-malic enzyme (ME) and NADPH-oxidase (*OsRbohB*). The *de novo* synthesis of OsRbohB and its trafficking to the plasma membrane contributes to iron- and ROS-dependent ferroptotic death in rice cells. Disease (susceptibility)-related cell death is ROS-dependent and iron-independent in the compatible Δ*Osmek2*–*M. oryzae* interaction. MEK, mitogen-activated protein kinase kinase; MPK, mitogen-activated protein kinase; NADP-ME, NADP-malic enzyme; NLR, nucleotide-binding leucine-rich repeat; PAMP, pathogen-associated molecular pattern; PRR, pattern recognition receptor; WRKY, tryptophan (W), arginine (R), lysine (K), tyrosine (Y) transcription factor.

Here, we combine our cumulative data to propose the following working model: OsMEK2-OsMPK1-OsWRKY90 signaling positively regulates iron- and ROS-dependent ferroptotic HR cell death in rice–*M. oryzae* interactions (Figure 12). The invasion of avirulent *M. oryzae* 007 into rice cells activates rice MAP kinases (*OsMEK2* and *OsMPK1*) via different hypothesized MAPK signaling pathways. The superoxide (O_2_^–^) produced from apoplastic oxygen (O_2_) can be converted to hydrogen peroxide (H_2_O_2_) by superoxide dismutase (SOD) (Figure 12; Marino et al., 2012; Kadota et al., 2015). Apoplastic ROS (H_2_O_2_) produced by plasma membrane-bound Rbohs during ETI migrates across the plasma membrane using aquaporin channels and into the cell (Bienert and Chaumont, 2014; Jwa and Hwang, 2017). The increased accumulation of iron (Fe^3+^) and lipid ROS triggers lipid peroxidation and subsequent ferroptotic cell death (Figure 12; Dixon et al., 2012; Stockwell et al., 2017; Dangol et al., 2019). The invasion of avirulent *M. oryzae* into rice cells activates rice MAP kinases (*OsMEK2* and *OsMPK1*) via different hypothesized MAPK signaling pathways. The perception of PAMPs or pathogen effectors via membrane-bound PRRs or NLRs, respectively, activates OsMAP kinase cascades in rice cells, as proposed previously (Jones and Dangl, 2006; Zipfel, 2008). MAP kinase kinases (MEKs) activate MAP kinases, which migrate from the cytoplasm to the nucleus and regulate transcriptional reprogramming (Morris, 2001; Ahlfors et al., 2004). OsMEK2 interacts with and phosphorylates downstream rice MAP kinase 1 (OsMPK1) and MAP kinase 6 (OsMPK6) in the cytoplasm (Singh et al., 2012). OsMPK1 moves from the cytoplasm into the nucleus to interact with the OsWRKY90 transcription factor (Figure 11; Singh et al., 2012). *OsMEK2* expression triggers OsMPK1-OsWRKY90 signaling pathways in the nucleus (Figure 11), which may lead to the upregulation of OsNADP-malic enzyme and rice NADPH-oxidase B (*OsRbohB*) (Figure 12). The MAPK-WRKY pathway activates Rbohs, leading to a prolonged and robust ROS burst (Adachi et al., 2015). The *de novo* synthesis of OsRbohB and its trafficking to the plasma membrane is involved in iron- and ROS-dependent ferroptotic death in rice cells. Rice MAP kinase 1 (OsMPK1) also may target OsWRKY90 to bind to specific sequences of some defense-related genes such as *OsPR-1b*. Disease (susceptibility)-related cell death is lipid ROS-dependent, but iron-independent, in the compatible rice–*M. oryzae* interaction (Figure 12). Iron accumulation may not mediate disease-related cell death in rice. Iron-independent disease-related cell death is likely a necrosis-type cell death that is distinct from ferroptotic cell death in incompatible rice–*M. oryzae* interactions. However, iron- and ROS-dependent ferroptotic cell death in rice is a generally regulated form of cell death that is common in incompatible rice–*M. oryzae* interactions (Dangol et al., 2019). Iron accumulation in ferroptotic cells may be harmful to the invaded hyphae of avirulent *M. oryzae*. Further research on the functions of this ROS-dependent disease-related cell death is required to determine how virulent *M. oryzae* infection suppresses ferroptotic cell death and induces disease-related cell death, which may be beneficial for pathogen growth *in planta*.

## Data Availability Statement

The original contributions presented in the study are included in the article/Supplementary Material, further inquiries can be directed to the corresponding author.

## Author Contributions

N-SJ designed the research. SD, NKN, RS, YC, JW, and H-GL carried out all the experiments. SD, NKN, BKH, and N-SJ analyzed the data and wrote the manuscript. BKH and N-SJ reviewed and edited the final manuscript. All authors read and agreed to the published version of the manuscript.

## Conflict of Interest

The authors declare that the research was conducted in the absence of any commercial or financial relationships that could be construed as a potential conflict of interest.

## Publisher’s Note

All claims expressed in this article are solely those of the authors and do not necessarily represent those of their affiliated organizations, or those of the publisher, the editors and the reviewers. Any product that may be evaluated in this article, or claim that may be made by its manufacturer, is not guaranteed or endorsed by the publisher.

## Footnotes

1
http://signal.salk.edu./cgi-bin/RiceGE

2
http://www.nics.go.kr

3
http://genebank.snu.ac.kr

## Supplementary Material

Supplementary Figure 1 Amino acid sequence alignment and phylogenetic tree of rice MAPKKs (OsMEKs) with Arabidopsis MAPKKs. **(A)** Amino acid sequence alignment of OsMEKs with Arabidopsis MAPKKs. Rice MAPKKs are aligned with Arabidopsis MAPKKs, which are categorized into four groups (Group A–D) using Clustal Omega (EMBL-EBI). The MAPKK active site [D(I/L/V)K] and conserved domain [S/T-X-S/T] are located between kinase subdomains VII and VIII. **(B)** Phylogenetic tree of OsMEKs with Arabidopsis MAPKKs was constructed using the neighbor-joining method based on Molecular Evolutionary Genetics Analysis Version 7.0 (MEGA7) (Kumar et al., 2016). Accession numbers of the plant MAPKKs are OsMEK1 (Os01g32660), OsMEK2 (Os06g05520), OsMEK3 (Os03g12390), OsMEK4 (Os02g46760), OsMEK5 (Os06g09190), OsMEK6 (Os02g54600), OsMEK7 (Os06g09180), OsMEK8 (Os06g27890), OsWNK1 (Os07g38530), AtMKK1 (At4g26070), AtMKK2 (At4g29810), AtMEK3 (NP_198860), AtMEK4 (At1g51660), AtMEK5 (At3g21220), AtMKK6 (At5g56580), AtMKK9 (At1g73500), and AtWNK9 (At3g04910).

Supplementary Figure 2 Amino acid sequence alignment of rice MAPKKs (OsMEKs) with Arabidopsis, tomato, and tobacco MEKs. Amino acid sequence of OsMEK2 was aligned with MAP kinase kinases of Arabidopsis, tomato, and tobacco using CLUSTAL OMEGA (EMBL-EBI). Accession numbers of the aligned plant MAPKKs are OsMEK1 (Os01g32660), OsMEK2 (Os06g05520), OsMEK3 (Os03g12390), OsMEK6 (Os02g54600), OsMEK8 (Os06g27890), AtMKK1 (At4g26070), AtMKK2 (At4g29810), AtMEK3 (NP_198860), AtMEK4 (At1g51660), AtMEK5 (At3g21220), SlMKK2 (NP_001234588), NtMEK2 (AF325168), and NbMEK2 (LOC107818847). Asterisks and dots at the bottom of sequences indicate identical and similar amino acids, respectively. Domain numbers (I∼XI) on the top of sequences indicate conserved subdomains. The conserved consensus motif (GXGXXG) in conserved subdomain I is boxed. The active motif [D(I/L/V)K] and the conserved consensus motif [S/TXXXXXS/T, serine(S)/threonine (T) residues] between conserved subdomains VII and VIII are shown in yellow.

Supplementary Figure 3 Circular phylogenetic tree of rice MAPKKs (OsMEKs) with Arabidopsis, tomato, and tobacco MAPKs. Phylogenetic tree was constructed using the neighbor-joining method based on Molecular Evolutionary Genetics Analysis Version 7.0 (MEGA7) (Kumar et al., 2016). Accession numbers of the plant MAPKKs are OsMEK1 (Os01g32660), OsMEK2 (Os06g05520), OsMEK3 (Os03g12390), OsMPK8a (Os06g27890), AtMEK3 (NP_198860), AtMEK4 (At1g51660), AtMEK5 (At3g21220), AtMKK1 (At4g26070), AtMKK2 (At4g29810), SlMKK1 (NP_001234744), SlMKK2 (NP_001234588), NtMEK2 (AF325168), NtNPK2 (BAA06731), and NbMEK2 (LOC107818847).

Supplementary Figure 4 Nucleotide sequences and deduced amino acid sequences of rice MAP kinase kinase 2 (OsMEK2) genomic DNA. Small letters represent nucleotide sequences of exons and introns. Capital letters represent deduced amino acid sequences of exons. Initiation and termination codons of the *OsMEK2* coding region are represented by asterisks. Exon-intron splice junctions (gt/ag) are represented by bold letters. Numbers at the right refer to nucleotide and amino acid (in parentheses) residue positions in the respective sequence.

Supplementary Figure 5 Avirulent *Magnaporthe oryzae* 007 infection causes susceptible responses in Δ*Osmek2* knock-out lines, but resistant responses in the wild-type rice DJ and *35S:OsMEK2* overexpression lines. Rice leaf sheaths were inoculated with a conidial suspension (4 × 10^5^ conidia mL^–1^). **(A)** Images of rice sheath epidermal cells infected with *M. oryzae* 007 (48 hpi). *M. oryzae* 007 grew well and produced invasive hyphae in the Δ*Osmek2* #2 and #4 knock-out plants, but induced hypersensitive cell death in wild-type (WT) rice DJ and 35S:*OsMEK2* #4 and #6 overexpression plants. Images were captured using a fluorescence microscope. hpi, hours post-inoculation. Scale bars = 20 μm. (B) Quantification of invasive hyphae (IH) and cell death (CD) in rice sheath cells infected with *M. oryzae* 007 (48 hpi). Results are presented as mean values ± SD; *n* = 4 leaf sheaths from different plants. Different letters above the bars indicate significantly different means (*P* < 0.05), as analyzed by Fisher’s protected least significant difference (LSD) test. IH, invasive hyphae; CD, cell death.

Supplementary Figure 6 Quantitative real-time RT-PCR analysis of time-course expression of defense-response genes *OsPR-1b*, *OsPAL1*, *OsAPX1*, and *OsAPX2* in wild-type (WT) rice (cultivar DJ), Δ*Osmek2* #2, and 35S:*OsMEK2* #4 plants during *Magnaporthe oryzae* 007 infection. Leaf sheaths of wild-type (cultivar DJ), Δ*Osmek2* #2, and 35S:*OsMEK2* #4 rice plants were sampled at different time points after inoculation, followed by total RNA extraction. Relative gene expression levels of defense-responsive genes *OsPR-1b* (Os01g28450), *OsPAL1* (Os04g43760), *OsAPX1* (*Os0.g17690*), and *OsAPX2* (*Os07g49400*) at each time point were obtained by normalizing the gene expression with respect to expression of the internal control *18S rRNA* gene. Data represent the means ± SD from three independent experiments. Different letters above the bars indicate significantly different means (*P* < 0.05) as analyzed by Fisher’s protected LSD test.

Supplementary Figure 7 Images of Prussian blue-stained and DAB-stained leaf sheath cells of wild-type (WT) rice (cultivar DJ) at different time points after erastin treatment. Rice DJ leaf sheaths were treated with mock (water) and 10 μM erastin. Images were taken using a fluorescence microscope (Zeiss equipped with Axioplan 2). Prussian blue and DAB staining in erastin-treated rice leaf sheaths did not detect ferric ion and ROS accumulation, respectively, at different time points after treatment. Experiments were repeated three times with similar results. hpt, hours post-treatment. Scale bars = 20 μm.

Supplementary Figure 8 Nucleotide sequences and deduced amino acid sequences of rice MAP kinase 1 (OsMPK1) genomic DNA. Small letters represent nucleotide sequences of exons and introns. Capital letters represent deduced amino acid sequences of exons. Initiation and termination codons of the *OsMPK1* coding region are represented by asterisks. Numbers at the right refer to nucleotide and amino acid (in parentheses) residue positions in the respective sequence.

Supplementary Figure 9 Amino acid sequence alignment of rice MAP kinase 1 (OsMPK1) with other plant MPKs. Amino acid sequence of OsMPK1 was aligned with plant MAP kinases using CLUSTAL OMEGA (EMBL-EBI). Accession numbers of the aligned plant MPKs are OsMPK1 (Os06g06090), OsMPK6 (Os10g38950), AtMPK6 (At2g43790), AtMPK3 (At3g45640), AtMPK4 (At4g01370), AtMPK11 (At1g01560), NbMKK1 (BAE95414), LeMPK1 (NP_001234011), LeMPK2 (NP_001234355), LeMPK3 (NP_001234360), and ZmMPK5 (PW05027).

Supplementary Figure 10 Phylogenetic tree of rice MAP kinase 1 (OsMPK1) with plant MPKs. Accession numbers: MPKs are OsMPK1 (Os06g06090), OsMPK6 (Os10g38950), AtMPK6 (At2g43790), AtMPK3 (At3g45640), AtMPK4 (At4g01370), AtMPK11 (At1g01560), NbMKK1 (BAE95414), LeMPK1 (NP_001234011), LeMPK2 (NP_001234355), LeMPK3 (NP_001234360), and ZmMPK5 (PW05027).

Supplementary Figure 11 Transcriptional analysis of *OsMPK1* expression in leaf sheath epidermal cells of wild-type (WT) rice (cultivar NB) and 35S:*OsMPK1* plants using qRT-PCR. Relative gene expression of *OsMPK1* was obtained by normalizing with respect to the expression of the internal control *OsUbiquitin* gene. Data represent the means ± SD from three independent experiments. Asterisks indicate statistically significant differences (Student’s *t*-test, *P* < 0.01).

Supplementary Table 1 Primers used in this study.
