# Supplementary material for: Mitogen-Activated Protein Kinase OsMEK2 and OsMPK1 Signaling Is Required for Ferroptotic Cell Death in Rice–Magnaporthe oryzae Interactions
Source: Front Plant Sci. 2021 Aug 2;12:710794. doi: 10.3389/fpls.2021.710794 (PMC8365360; doi:10.3389/fpls.2021.710794)
Supplement: Supplementary Figure 1 — Amino acid sequence alignment and phylogenetic tree of rice MAPKKs (OsMEKs) with Arabidopsis MAPKKs. (A) Amino acid sequence alignment of OsMEKs with Arabidopsis MAPKKs. Rice MAPKKs are aligned with Arabidopsis MAPKKs, which are categorized into four groups (Group A–D) using Clustal Omega (EMBL-EBI). The MAPKK active site [D(I/L/V)K] and conserved domain [S/T-X-S/T] are located between kinase subdomains VII and VIII. (B) Phylogenetic tree of OsMEKs with Arabidopsis MAPKKs was constructed using the neighbor-joining method based on Molecular Evolutionary Genetics Analysis Version 7.0 (MEGA7) (Kumar et al., 2016). Accession numbers of the plant MAPKKs are OsMEK1 (Os01g32660), OsMEK2 (Os06g05520), OsMEK3 (Os03g12390), OsMEK4 (Os02g46760), OsMEK5 (Os06g09190), OsMEK6 (Os02g54600), OsMEK7 (Os06g09180), OsMEK8 (Os06g27890), OsWNK1 (Os07g38530), AtMKK1 (At4g26070), AtMKK2 (At4g29810), AtMEK3 (NP_198860), AtMEK4 (At1g51660), AtMEK5 (At3g21220), AtMKK6 (At5g56580), AtMKK9 (At1g73500), and AtWNK9 (At3g04910). [file Presentation_1.PPT]

## Slide 1
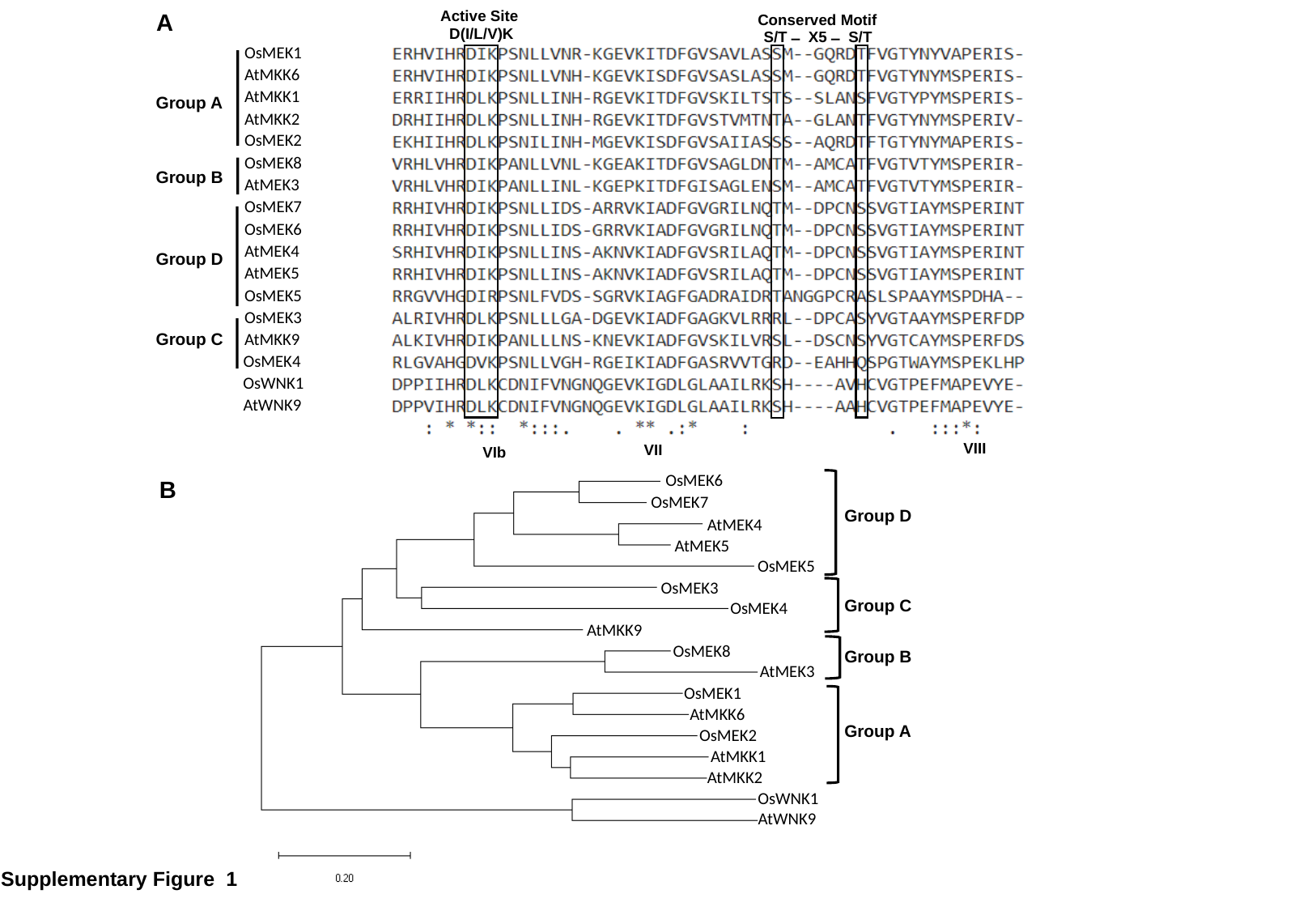

Active Site
D(I/L/V)K
Conserved Motif
S/T ̶ X5 ̶ S/T
VIII
Group A
Group B
Group D
Group C
VII
VIb
OsMEK1
AtMKK6
AtMKK1
AtMKK2
OsMEK2
OsMEK8
AtMEK3
OsMEK7
OsMEK6
AtMEK4
AtMEK5
OsMEK5
OsMEK3
AtMKK9
OsMEK4
OsWNK1
AtWNK9
A
OsMEK6
Group D
Group C
Group B
Group A
OsMEK7
AtMEK4
AtMEK5
OsMEK5
OsMEK3
OsMEK4
AtMKK9
OsMEK8
AtMEK3
OsMEK1
AtMKK6
OsMEK2
AtMKK1
AtMKK2
OsWNK1
AtWNK9
 B
Supplementary Figure 1

## Slide 2
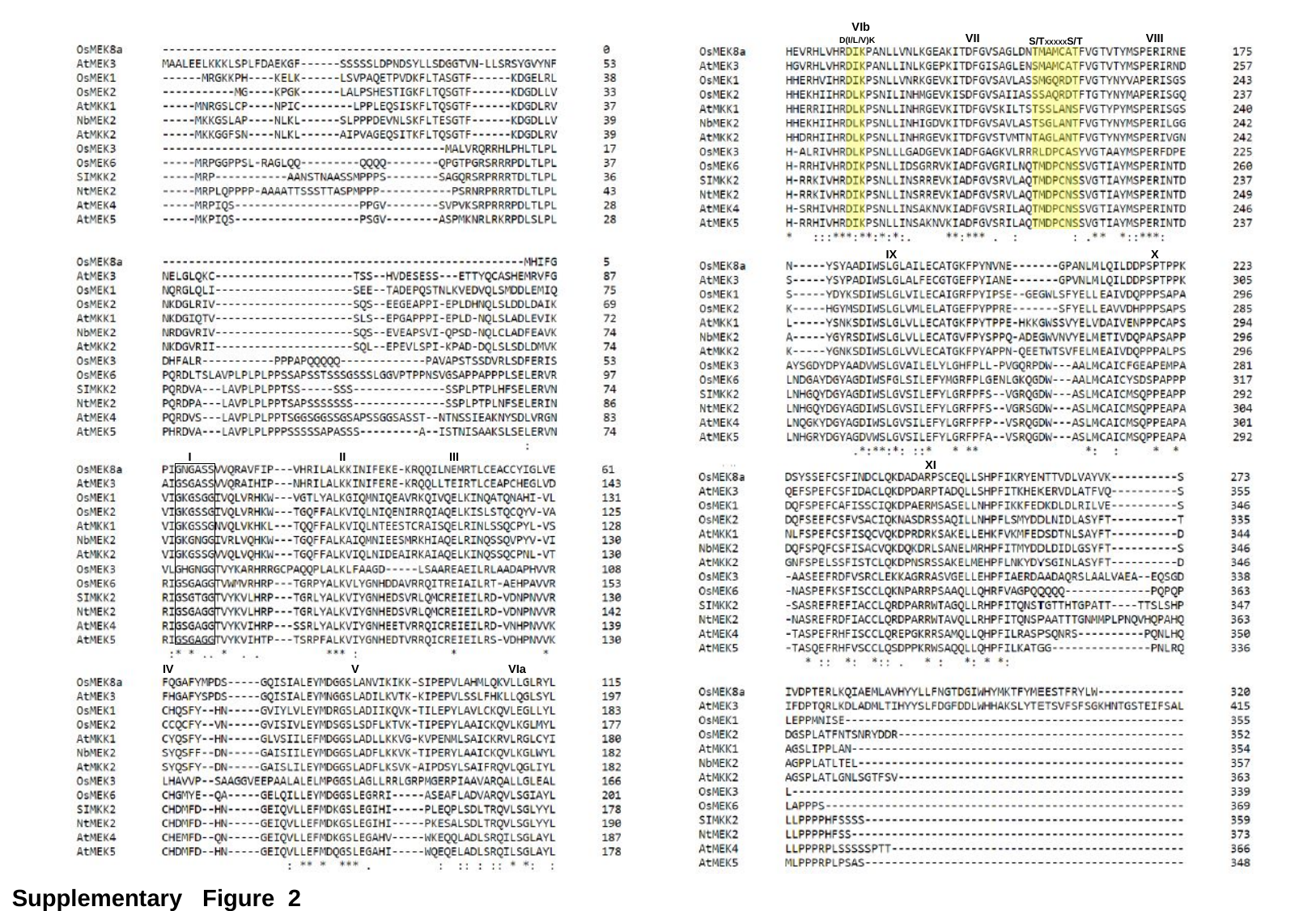

VIb
VII
VIII
S/TXXXXXS/T
D(I/L/V)K
IX
X
XI
I
II
III
IV
V
VIa
Supplementary Figure 2

## Slide 3
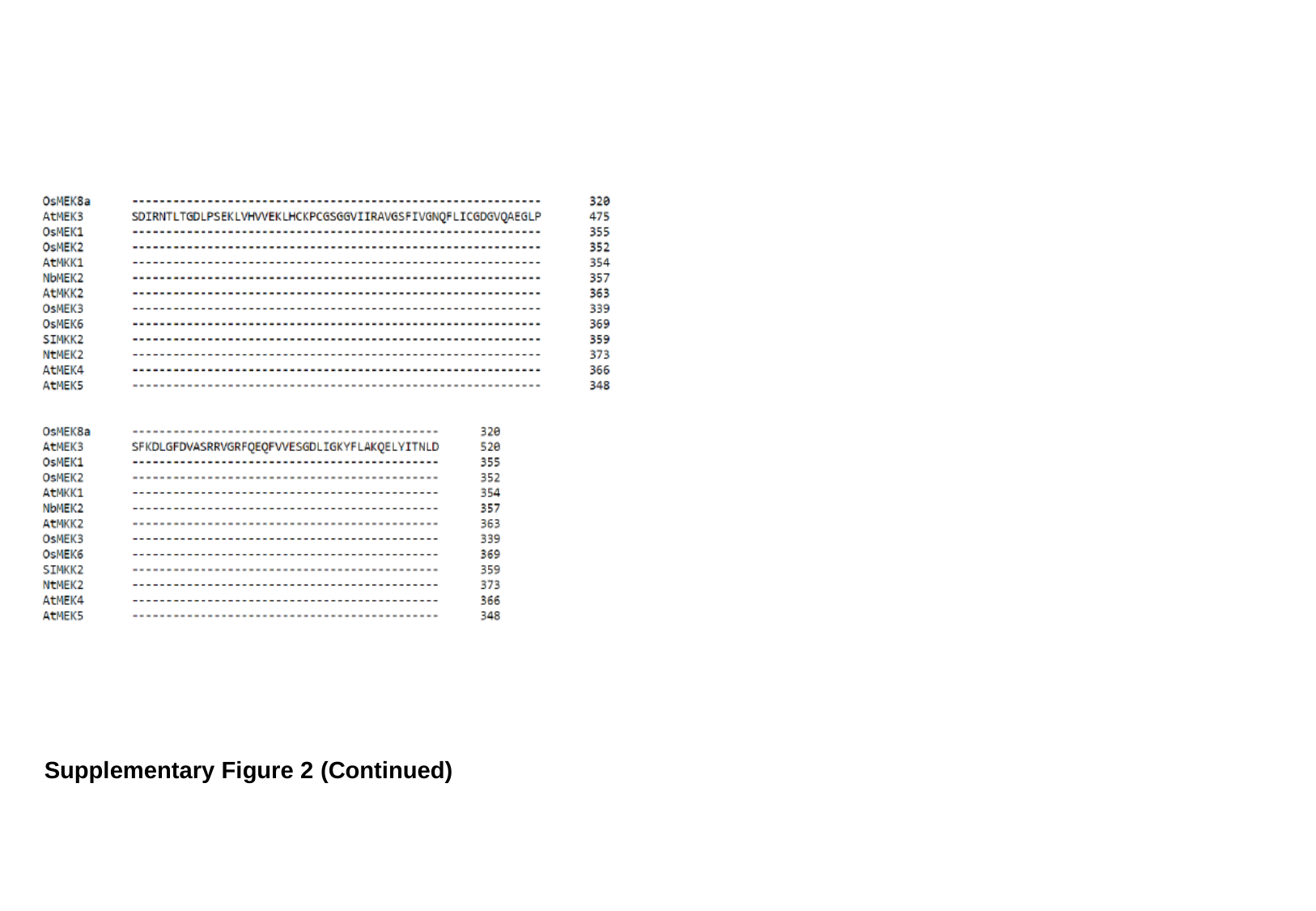

Supplementary Figure 2 (Continued)

## Slide 4
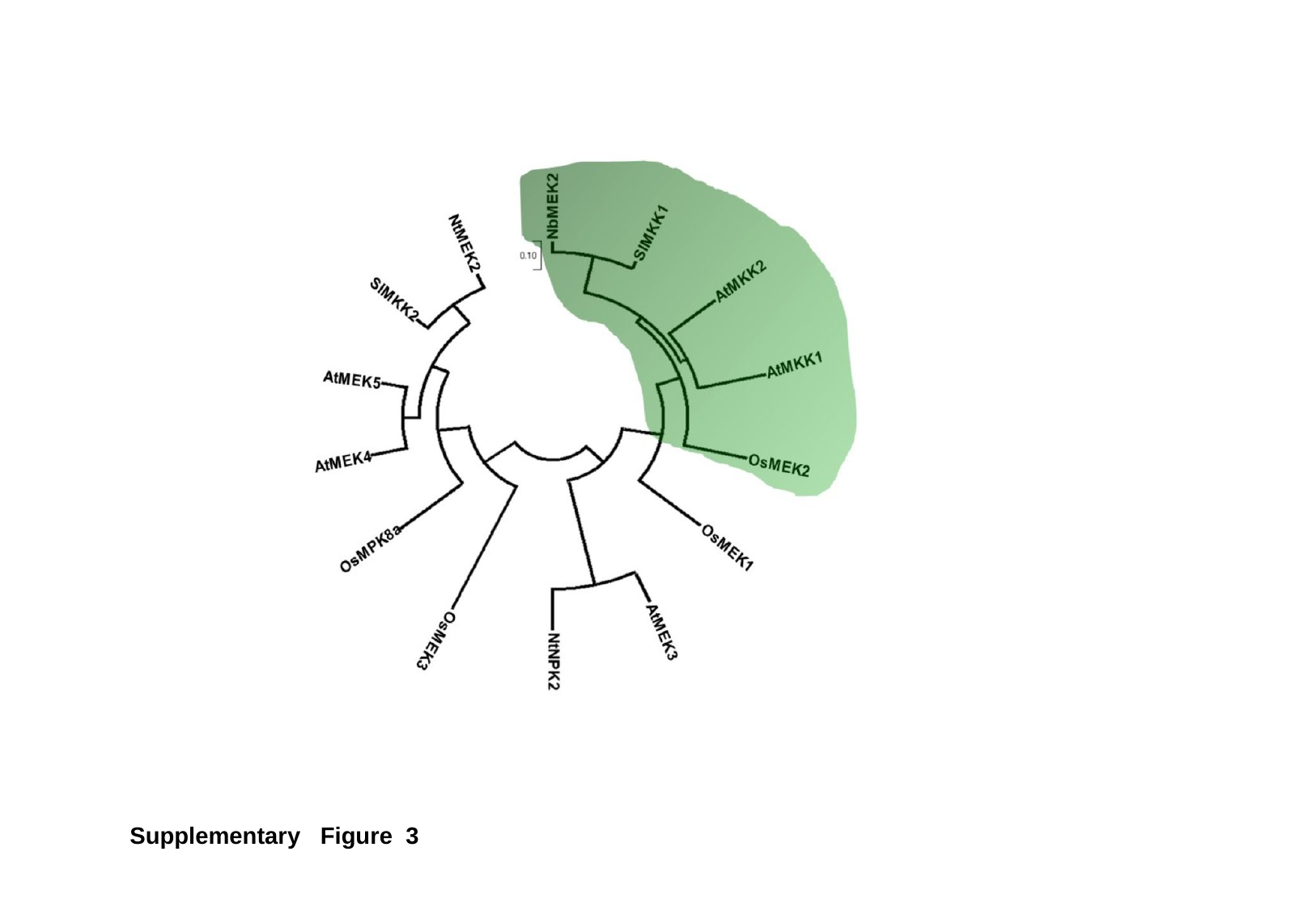

Supplementary Figure 3

## Slide 5
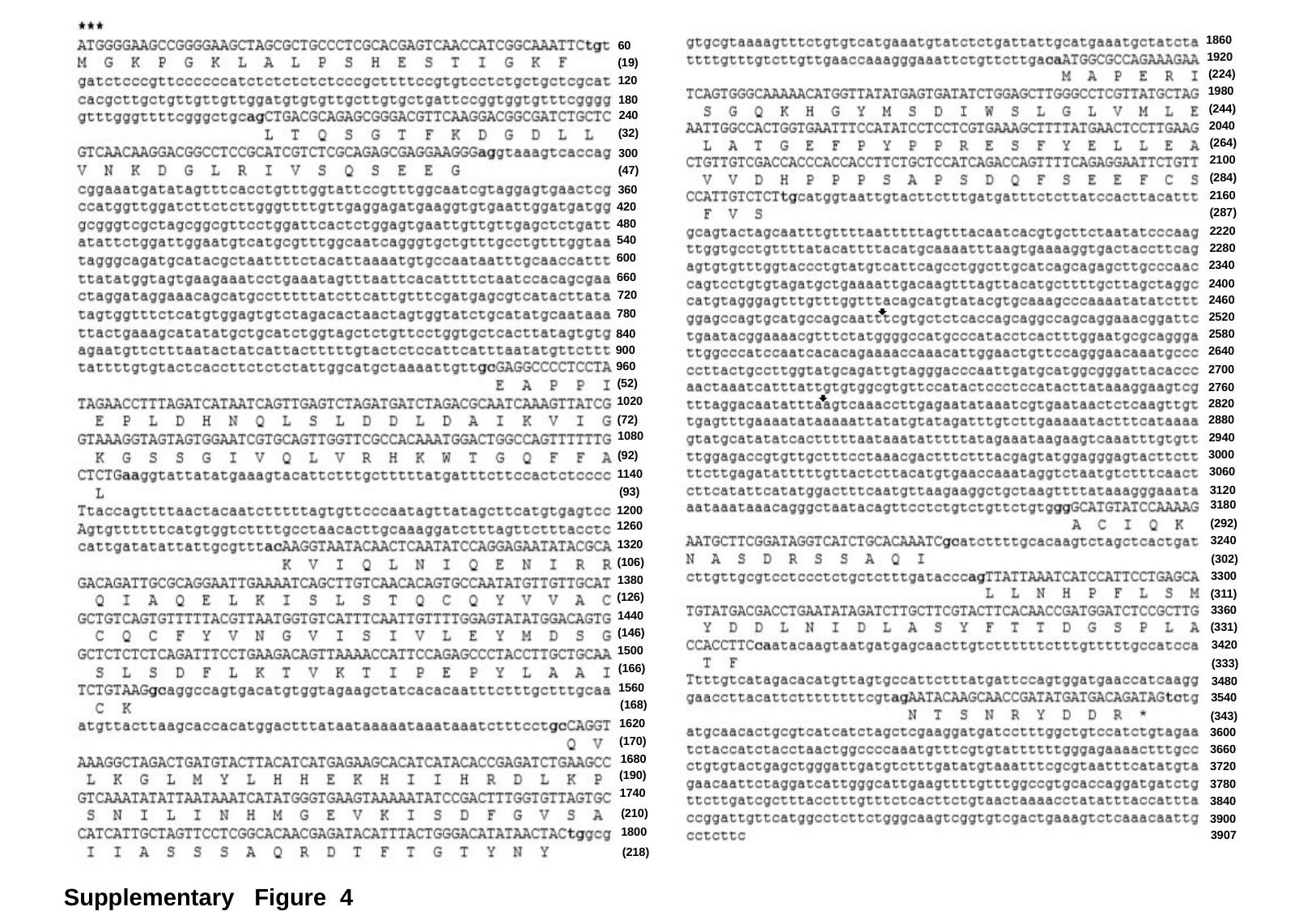

60
(19)
120
180
240
(32)
300
(47)
360
420
480
540
600
660
720
780
840
900
960
(52)
1020
(72)
1080
(92)
1140
(93)
1200
1260
1320
(106)
1380
(126)
1440
(146)
1500
(166)
1560
(168)
1620
(170)
1680
(190)
1740
(210)
1800
(218)
1860
1920
(224)
1980
(244)
2040
(264)
2100
(284)
2160
(287)
2220
2280
2340
2400
2460
2520
2580
2640
2700
2760
2820
2880
2940
3000
3060
3120
3180
(292)
3240
(302)
3300
(311)
3360
(331)
3420
(333)
3480
3540
(343)
3600
3660
3720
3780
3840
3900
3907
Supplementary Figure 4

## Slide 6
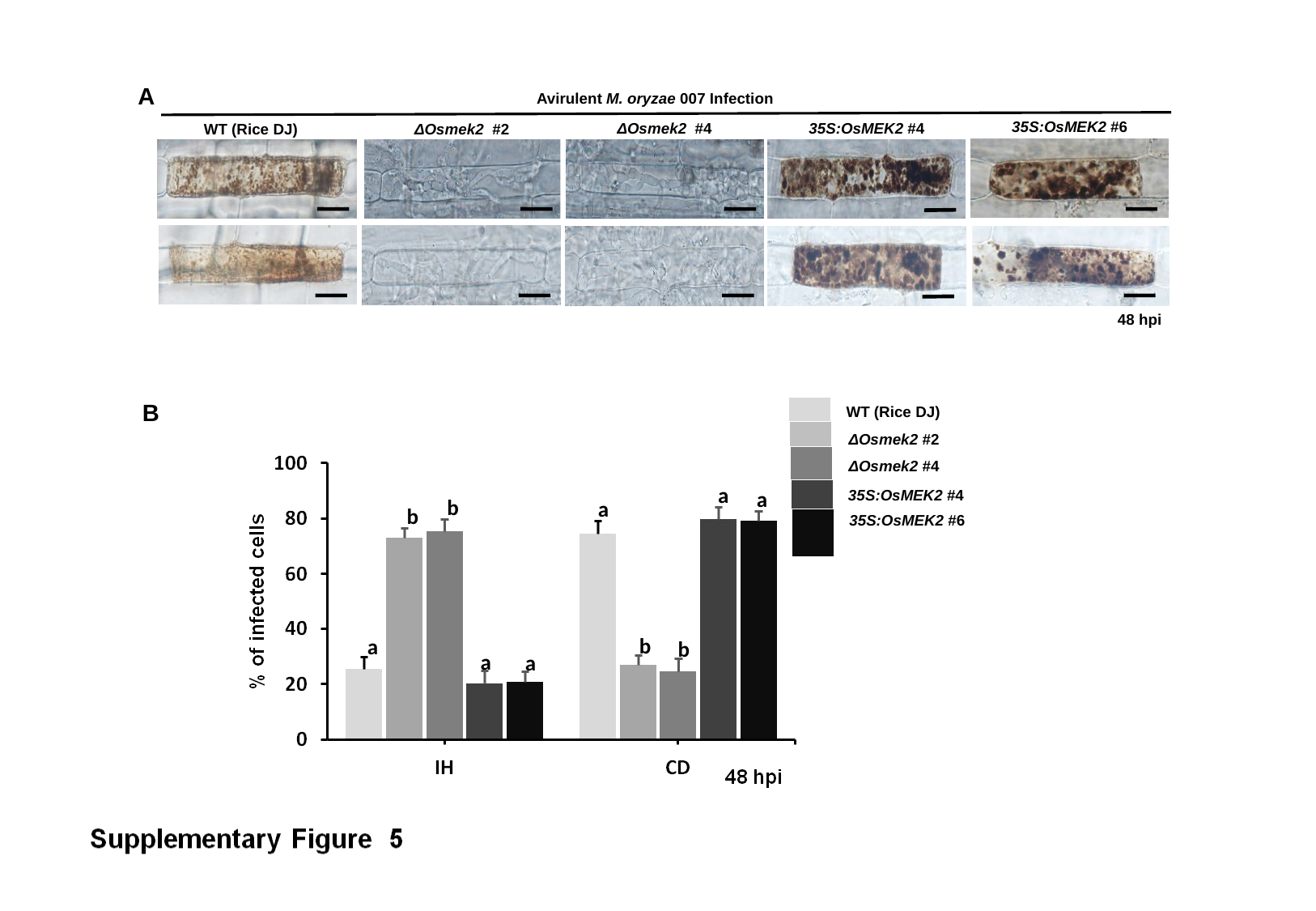

A
Avirulent M. oryzae 007 Infection
35S:OsMEK2 #6
ΔOsmek2 #4
35S:OsMEK2 #4
WT (Rice DJ)
ΔOsmek2 #2
48 hpi
B
WT (Rice DJ)
ΔOsmek2 #2
ΔOsmek2 #4
35S:OsMEK2 #4
35S:OsMEK2 #6
| |
| --- |
a
a
b
a
b
b
a
b
a
a
| |
| --- |
| |
| --- |
| |
| --- |
| |
| --- |

## Slide 7
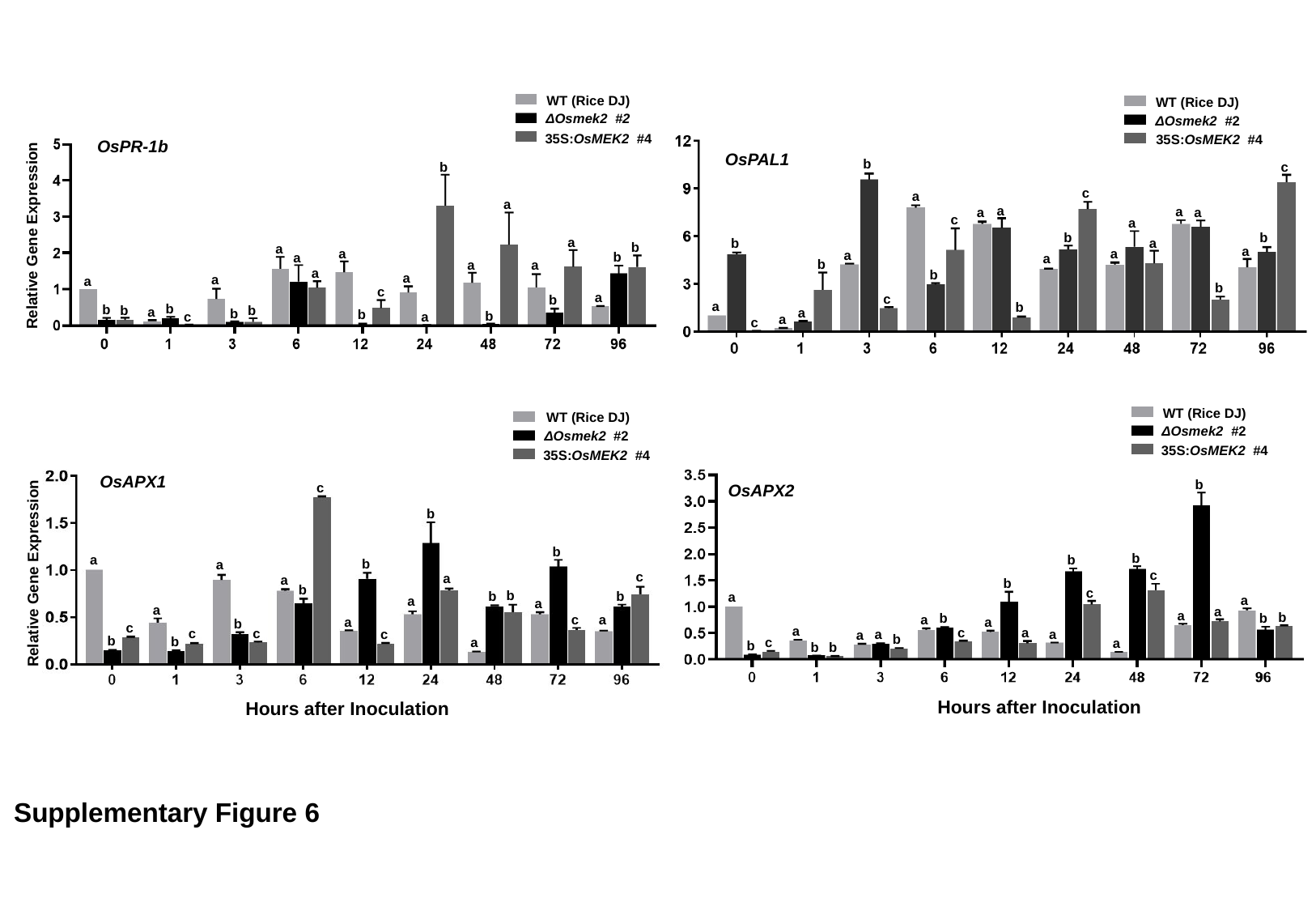

OsPR-1b
OsPAL1
Relative Gene Expression
WT (Rice DJ)
ΔOsmek2 #2
35S:OsMEK2 #4
WT (Rice DJ)
ΔOsmek2 #2
35S:OsMEK2 #4
b
b
c
c
a
a
a
a
a
a
c
a
b
b
a
b
a
b
a
a
a
a
a
b
a
a
b
a
a
a
b
a
a
a
b
c
a
c
b
a
b
b
b
b
b
a
a
b
b
b
c
a
a
c
WT (Rice DJ)
ΔOsmek2 #2
35S:OsMEK2 #4
WT (Rice DJ)
ΔOsmek2 #2
35S:OsMEK2 #4
OsAPX1
OsAPX2
Relative Gene Expression
b
c
b
b
b
a
b
b
a
c
c
a
a
b
b
c
b
b
b
a
a
a
a
a
a
a
b
b
b
c
a
a
a
a
b
c
a
c
a
c
c
c
a
a
a
b
b
b
a
c
a
b
b
b
a
Hours after Inoculation
Hours after Inoculation
Supplementary Figure 6

## Slide 8
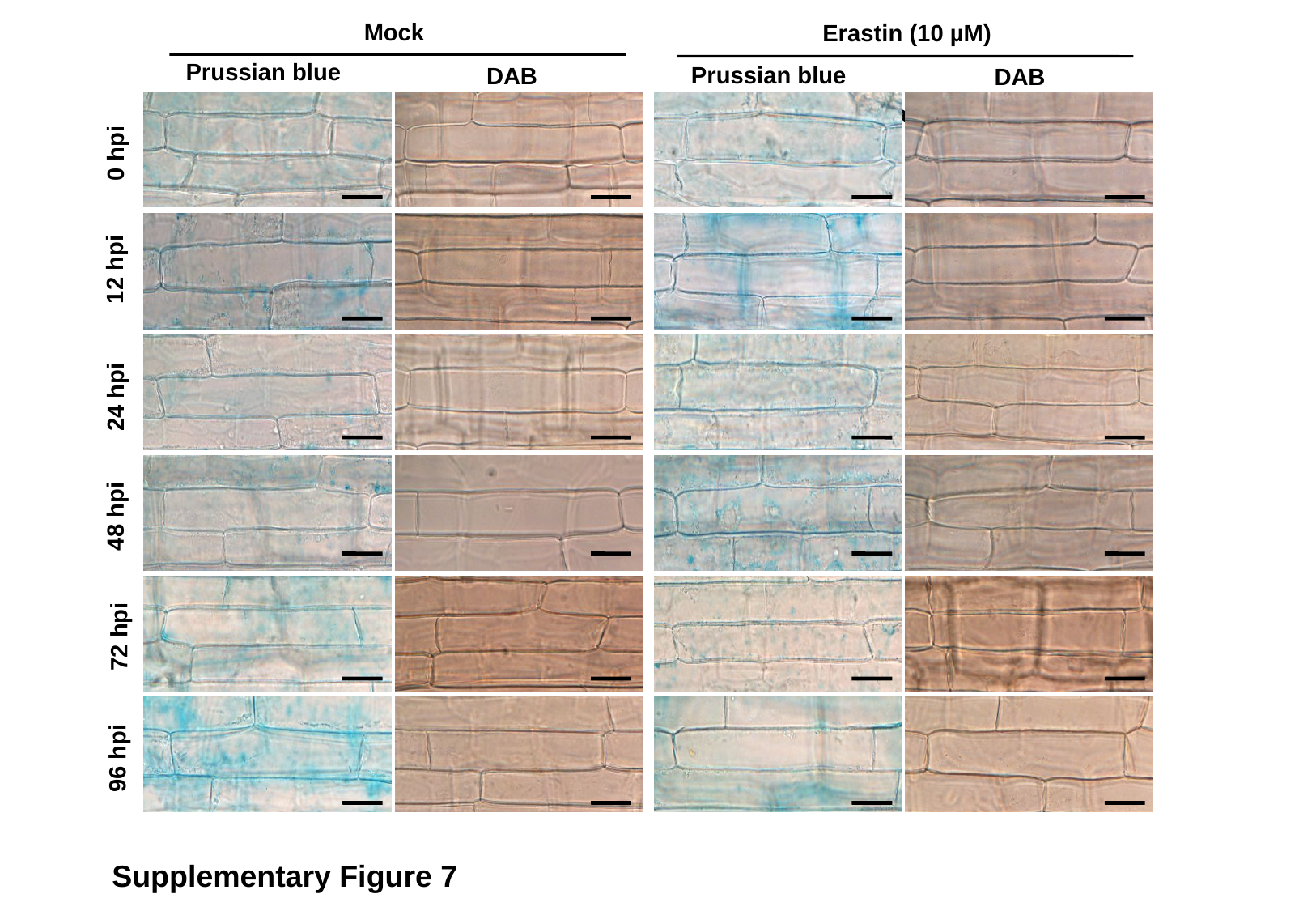

Mock
Prussian blue
Prussian blue
DAB
DAB
Erastin (
0 hpi
12 hpi
24 hpi
48 hpi
72 hpi
96 hpi
Erastin (10 µM)
Supplementary Figure 7

## Slide 9
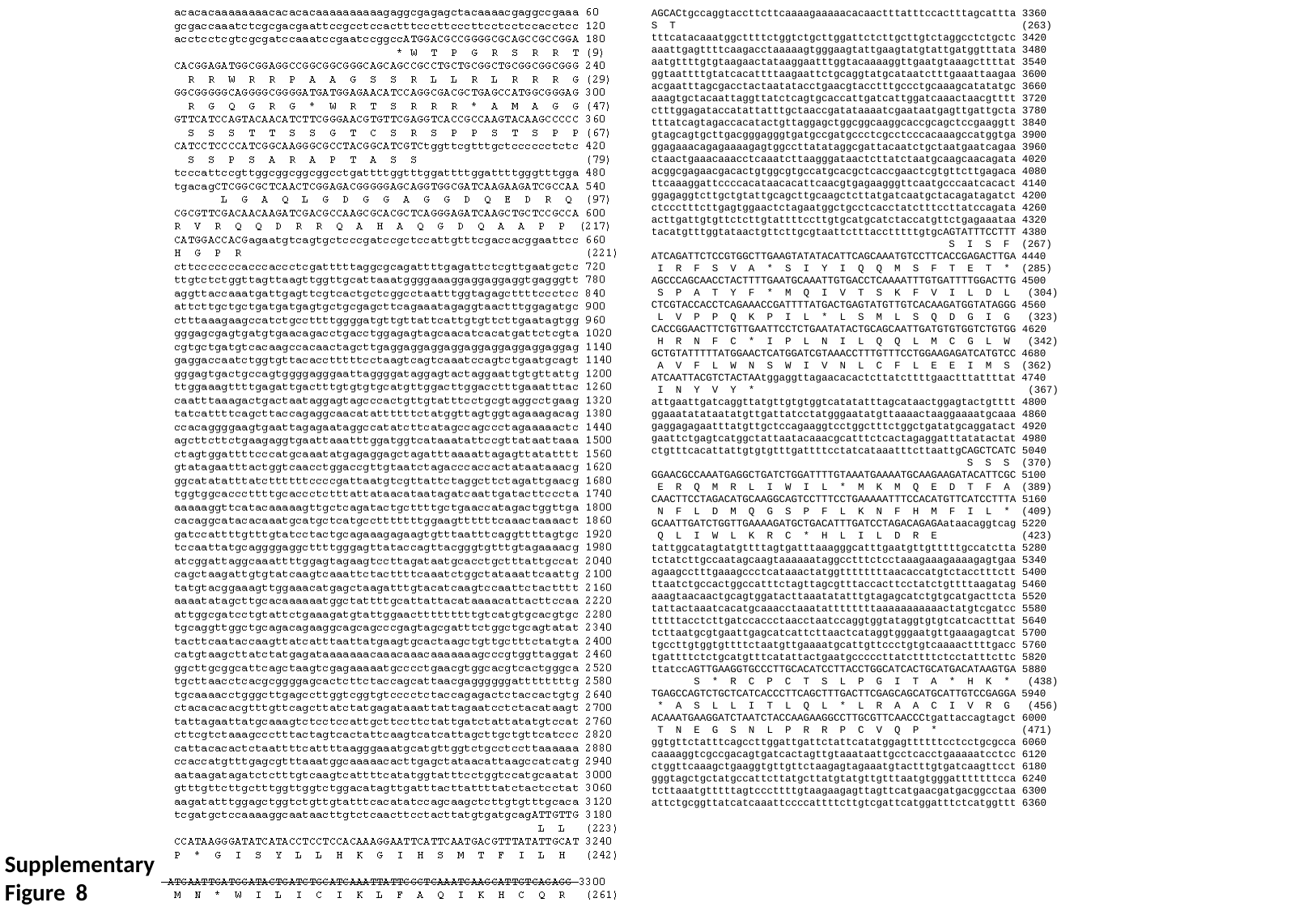

AGCACtgccaggtaccttcttcaaaagaaaaacacaactttatttccactttagcattta 3360
 S T (263)
 tttcatacaaatggcttttctggtctgcttggattctcttgcttgtctaggcctctgctc 3420
 aaattgagttttcaagacctaaaaagtgggaagtattgaagtatgtattgatggtttata 3480
 aatgttttgtgtaagaactataaggaatttggtacaaaaggttgaatgtaaagcttttat 3540
 ggtaattttgtatcacattttaagaattctgcaggtatgcataatctttgaaattaagaa 3600
 acgaatttagcgacctactaatatacctgaacgtacctttgccctgcaaagcatatatgc 3660
 aaagtgctacaattaggttatctcagtgcaccattgatcattggatcaaactaacgtttt 3720
 ctttggagataccatattatttgctaaccgatataaaatcgaataatgagttgattgcta 3780
 tttatcagtagaccacatactgttaggagctggcggcaaggcaccgcagctccgaaggtt 3840
 gtagcagtgcttgacgggagggtgatgccgatgccctcgcctcccacaaagccatggtga 3900
 ggagaaacagagaaaagagtggccttatataggcgattacaatctgctaatgaatcagaa 3960
 ctaactgaaacaaacctcaaatcttaagggataactcttatctaatgcaagcaacagata 4020
 acggcgagaacgacactgtggcgtgccatgcacgctcaccgaactcgtgttcttgagaca 4080
 ttcaaaggattccccacataacacattcaacgtgagaagggttcaatgcccaatcacact 4140
 ggagaggtcttgctgtattgcagcttgcaagctcttatgatcaatgctacagatagatct 4200
 ctccctttcttgagtggaactctagaatggctgcctcacctatctttccttatccagata 4260
 acttgattgtgttctcttgtattttccttgtgcatgcatctaccatgttctgagaaataa 4320
 tacatgtttggtataactgttcttgcgtaattctttacctttttgtgcAGTATTTCCTTT 4380
 S I S F (267)
 ATCAGATTCTCCGTGGCTTGAAGTATATACATTCAGCAAATGTCCTTCACCGAGACTTGA 4440
 I R F S V A * S I Y I Q Q M S F T E T * (285)
 AGCCCAGCAACCTACTTTTGAATGCAAATTGTGACCTCAAAATTTGTGATTTTGGACTTG 4500
 S P A T Y F * M Q I V T S K F V I L D L (304)
 CTCGTACCACCTCAGAAACCGATTTTATGACTGAGTATGTTGTCACAAGATGGTATAGGG 4560
 L V P P Q K P I L * L S M L S Q D G I G (323)
 CACCGGAACTTCTGTTGAATTCCTCTGAATATACTGCAGCAATTGATGTGTGGTCTGTGG 4620
 H R N F C * I P L N I L Q Q L M C G L W (342)
 GCTGTATTTTTATGGAACTCATGGATCGTAAACCTTTGTTTCCTGGAAGAGATCATGTCC 4680
 A V F L W N S W I V N L C F L E E I M S (362)
 ATCAATTACGTCTACTAAtggaggttagaacacactcttatcttttgaactttattttat 4740
 I N Y V Y * (367)
 attgaattgatcaggttatgttgtgtggtcatatatttagcataactggagtactgtttt 4800
 ggaaatatataatatgttgattatcctatgggaatatgttaaaactaaggaaaatgcaaa 4860
 gaggagagaatttatgttgctccagaaggtcctggctttctggctgatatgcaggatact 4920
 gaattctgagtcatggctattaatacaaacgcatttctcactagaggatttatatactat 4980
 ctgtttcacattattgtgtgtttgattttcctatcataaatttcttaattgCAGCTCATC 5040
 S S S (370)
 GGAACGCCAAATGAGGCTGATCTGGATTTTGTAAATGAAAATGCAAGAAGATACATTCGC 5100
 E R Q M R L I W I L * M K M Q E D T F A (389)
 CAACTTCCTAGACATGCAAGGCAGTCCTTTCCTGAAAAATTTCCACATGTTCATCCTTTA 5160
 N F L D M Q G S P F L K N F H M F I L * (409)
 GCAATTGATCTGGTTGAAAAGATGCTGACATTTGATCCTAGACAGAGAataacaggtcag 5220
 Q L I W L K R C * H L I L D R E (423)
 tattggcatagtatgttttagtgatttaaagggcatttgaatgttgtttttgccatctta 5280
 tctatcttgccaatagcaagtaaaaaataggcctttctcctaaagaaagaaaagagtgaa 5340
 agaagcctttgaaagccctcataaactatggttttttttaacaccatgtctacctttctt 5400
 ttaatctgccactggccatttctagttagcgtttaccacttcctatctgttttaagatag 5460
 aaagtaacaactgcagtggatacttaaatatatttgtagagcatctgtgcatgacttcta 5520
 tattactaaatcacatgcaaacctaaatattttttttaaaaaaaaaaactatgtcgatcc 5580
 tttttacctcttgatccaccctaacctaatccaggtggtataggtgtgtcatcactttat 5640
 tcttaatgcgtgaattgagcatcattcttaactcataggtgggaatgttgaaagagtcat 5700
 tgccttgtggtgttttctaatgttgaaaatgcattgttccctgtgtcaaaacttttgacc 5760
 tgattttctctgcatgtttcatattactgaatgcccccttatcttttctcctatttcttc 5820
 ttatccAGTTGAAGGTGCCCTTGCACATCCTTACCTGGCATCACTGCATGACATAAGTGA 5880
 S * R C P C T S L P G I T A * H K * (438)
 TGAGCCAGTCTGCTCATCACCCTTCAGCTTTGACTTCGAGCAGCATGCATTGTCCGAGGA 5940
 * A S L L I T L Q L * L R A A C I V R G (456)
 ACAAATGAAGGATCTAATCTACCAAGAAGGCCTTGCGTTCAACCCtgattaccagtagct 6000
 T N E G S N L P R R P C V Q P * (471)
 ggtgttctatttcagccttggattgattctattcatatggagttttttcctcctgcgcca 6060
 caaaaggtcgccgacagtgatcactagttgtaaataattgcctcacctgaaaaatcctcc 6120
 ctggttcaaagctgaaggtgttgttctaagagtagaaatgtactttgtgatcaagttcct 6180
 gggtagctgctatgccattcttatgcttatgtatgttgtttaatgtgggatttttttcca 6240
 tcttaaatgtttttagtcccttttgtaagaagagttagttcatgaacgatgacggcctaa 6300
 attctgcggttatcatcaaattccccattttcttgtcgattcatggatttctcatggttt 6360
Supplementary Figure 8

## Slide 10
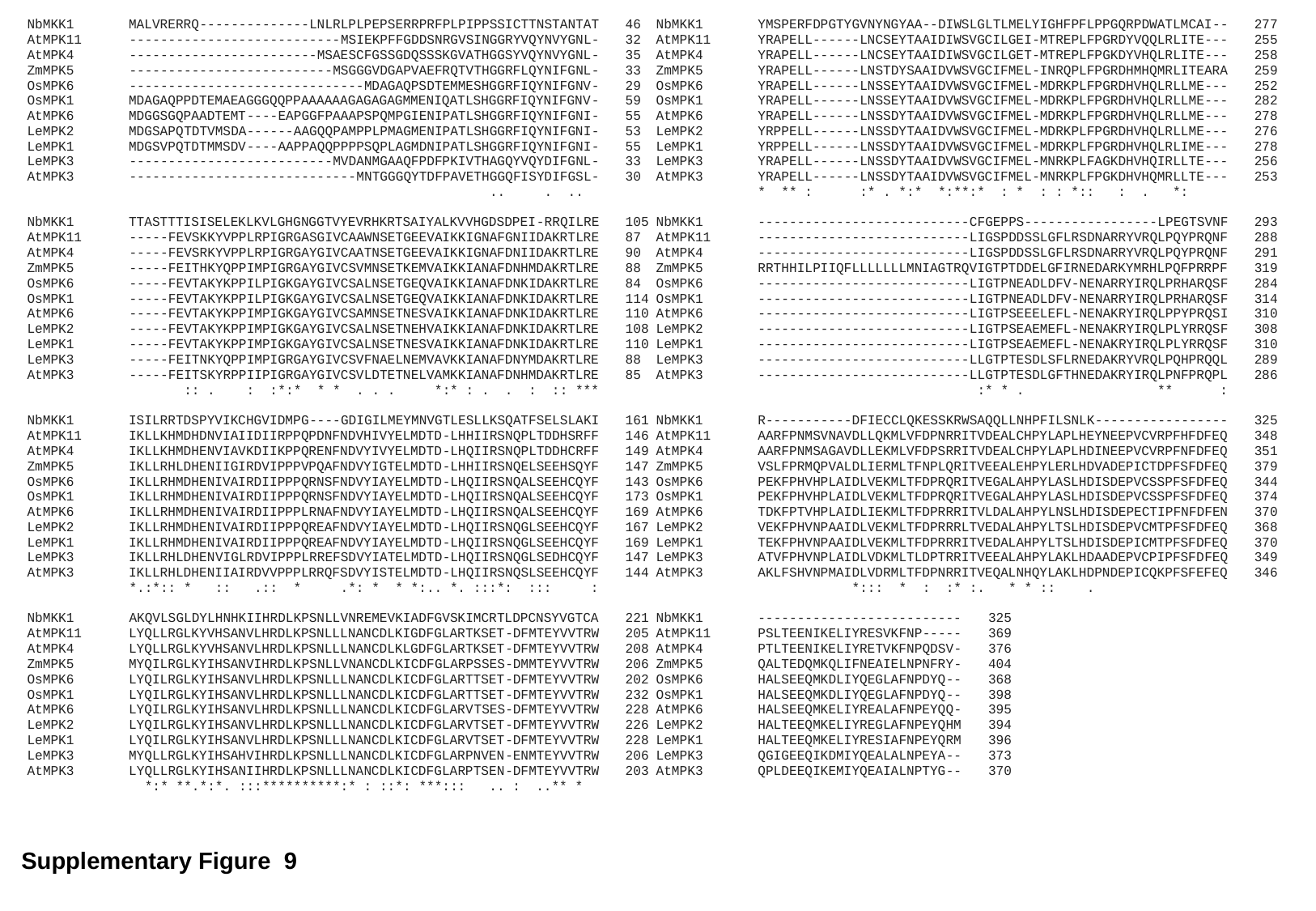

Supplementary Figure 9

## Slide 11
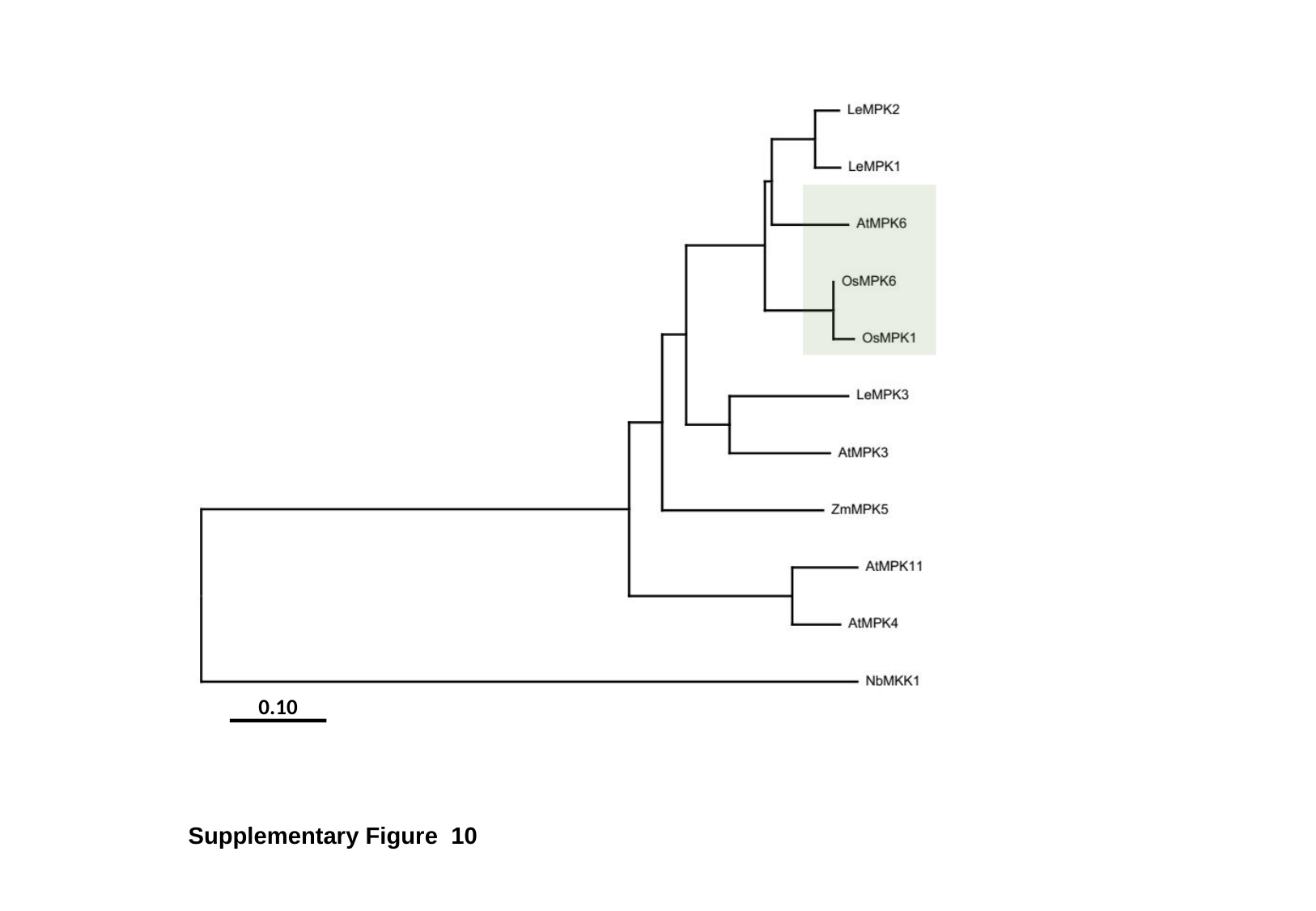

0.10
Supplementary Figure 10

## Slide 12
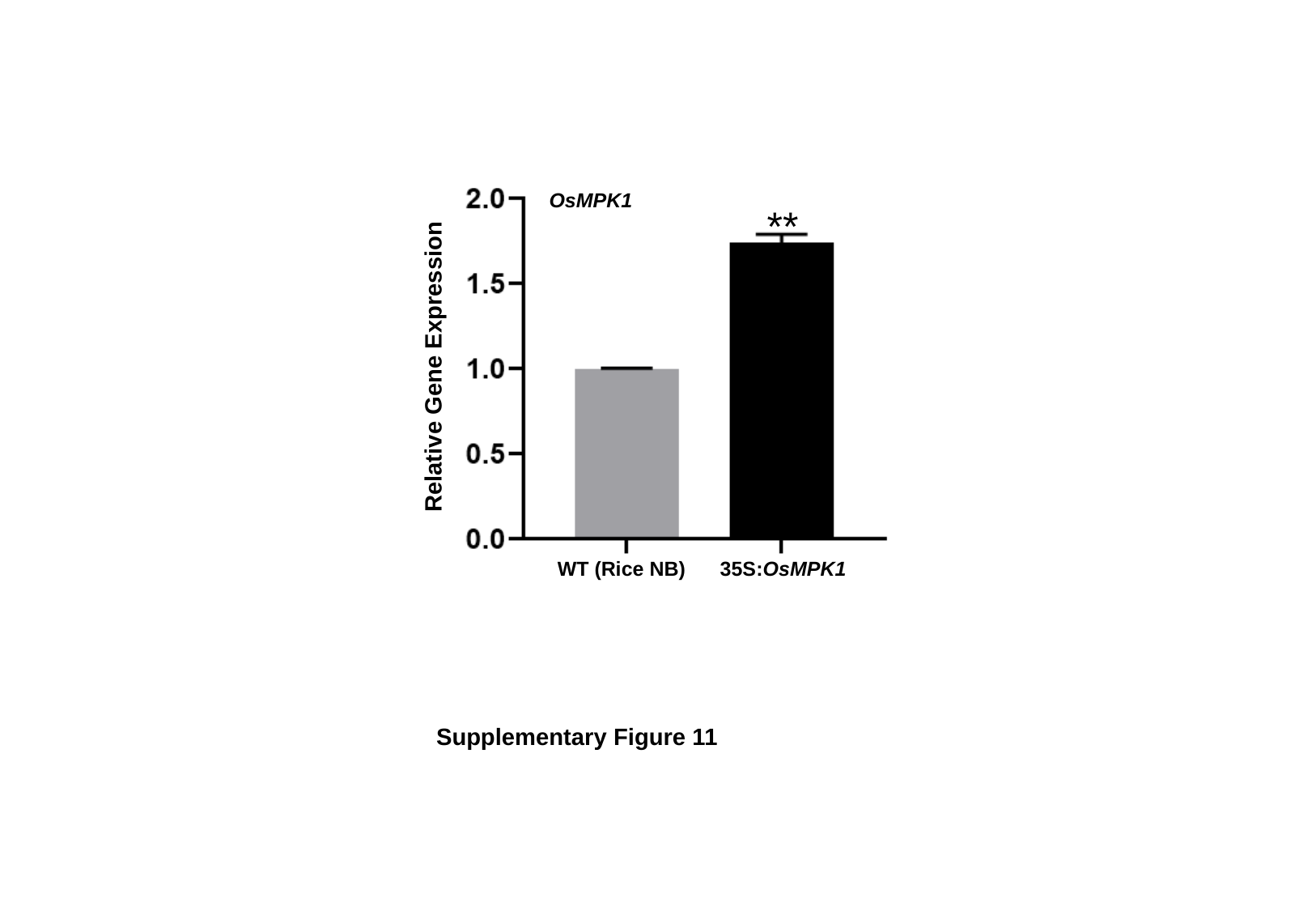

OsMPK1
**
Relative Gene Expression
WT (Rice NB)
35S:OsMPK1
Supplementary Figure 11
